# RuNi Nanoparticles Embedded in N‐Doped Carbon Nanofibers as a Robust Bifunctional Catalyst for Efficient Overall Water Splitting

**DOI:** 10.1002/advs.201901833

**Published:** 2019-11-18

**Authors:** Meixuan Li, Huiyuan Wang, Wendong Zhu, Weimo Li, Ce Wang, Xiaofeng Lu

**Affiliations:** ^1^ Alan G. MacDiarmid Institute College of Chemistry Jilin University Changchun 130012 P. R. China; ^2^ Key Laboratory of Automobile Materials of Ministry of Education and School of Materials Science and Engineering Nanling Campus Jilin University No. 5988 Renmin Street Changchun 130025 P. R. China; ^3^ International Center of Future Science Jilin University Changchun 130012 P. R. China

**Keywords:** bifunctional electrocatalysts, electrospinning, nitrogen‐doped carbon nanofibers, overall water splitting, RuNi nanoparticles

## Abstract

Developing high‐performance, low‐cost, and robust bifunctional electrocatalysts for overall water splitting is extremely indispensable and challenging. It is a promising strategy to couple highly active precious metals with transition metals as efficient electrocatalysts, which can not only effectively reduce the cost of the preparation procedure, but also greatly improve the performance of catalysts through a synergistic effect. Herein, Ru and Ni nanoparticles embedded within nitrogen‐doped carbon nanofibers (RuNi‐NCNFs) are synthesized via a simple electrospinning technology with a subsequent carbonization process. The as‐formed RuNi‐NCNFs represent excellent Pt‐like electrocatalytic activity for the hydrogen evolution reaction (HER) in both alkaline and acidic conditions. Furthermore, the RuNi‐NCNFs also exhibit an outstanding oxygen evolution reaction (OER) activity with an overpotential of 290 mV to achieve a current density of 10 mA cm^−2^ in alkaline electrolyte. Strikingly, owing to both the HER and OER performance, an electrolyzer with RuNi‐NCNFs as both the anode and cathode catalysts requires only a cell voltage of 1.564 V to drive a current density of 10 mA cm^−2^ in an alkaline medium, which is lower than the benchmark of Pt/C||RuO_2_ electrodes. This study opens a novel avenue toward the exploration of high efficient but low‐cost electrocatalysts for overall water splitting.

## Graphical abstract

A facile strategy based on electrospinning and a postcarbonization process is demonstrated to prepare carbon nanofibers incorporating Ru and Ni nanoparticles, which exhibits admirable Pt‐like hydrogen evolution reaction activity and superior oxygen evolution reaction performance. The electrolyzer with this hybrid as both anode and cathode displays a remarkable electrocatalytic activity and outstanding long‐term durability, which outperforms the commercial Pt/C||RuO_2_ electrocatalyst.

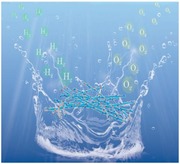

Hydrogen (H_2_) is a clean, renewable energy carrier and a promising alternative to fossil fuels.^1^, ^2^ The sustainable hydrogen production from electrocatalytic water splitting is one of the most efficient solutions at present, which is divided into two half reactions, namely anode oxygen evolution reaction (OER) and cathode hydrogen evolution reaction (HER). However, these reactions with sluggish kinetics and high overpotential are usually necessary to be carried out in the presence of highly efficient electrocatalysts to overcome the energy barriers.^3^, ^4^, ^5^ Currently, noble platinum‐based catalysts are recognized as the state‐of‐the‐art HER electrocatalysts, but their comprehensive application is restricted owing to their scarcity and valuableness.^6^, ^7^ Although nonprecious metal such as transition metal (TM)‐based materials have been extensively employed as robust HER catalysts, the greatest obstacle for the utilization of TM materials to date is their lower activity compared with that of Pt‐based catalysts and they are also vulnerable to strong acid and alkali corrosion.^8^, ^9^ The same impediments are still inevitable for TM‐based OER catalysts, as a higher overpotential is usually required to drive OER with a lower energy conversion efficiency. At present, the pure TM‐based catalysts can no longer reach the demand of substituting the noble‐metal‐based electrocatalysts. Therefore, it is significantly imperative to develop inexpensive bifunctional electrocatalysts in the same electrolyte for both HER and OER that are comparable in catalytic activities to the state‐of‐the‐art catalysts, e.g., Pt/C for HER and RuO_2_ or IrO_2_ for OER.

Recent studies have explored that ruthenium (Ru) shows a similar bond strength with hydrogen (≈65 kcal mol^−1^) and possesses more economic advantages than Pt‐based metals, which is a promising alternative to Pt.^10^, ^11^, ^12^ It has been generally considered that Ru‐based materials have aroused widespread attention owing to its promising activities for the HER and OER as well as the favorable stability under extreme conditions.^13^ However, the activity of the individual Ru nanomaterials does not achieve the desired electrocatalytic performance. It is shown that the content of precious metals is even reduced by an order of magnitude by chemical coupling with other TMs, which is a primary method to fabricate highly efficient catalysts with superior cost competitiveness.^14^, ^15^ Among TM‐based materials, Ni‐based catalysts displayed optimum activity in alkaline media, due to their optimum OH–Ni^2+δ^ (0 ≤ δ ≤ 1.5) bond strength that is conducive to the adsorption of intermediate.^16^ In addition, it can be observed from the volcanic correlation diagram between the activity of HER and the strength of hydrogen–metal bond that the binding between Ni and H is weak, while Ru strongly binds H.^17^, ^18^ The chemical coupling of Ru with Ni can reduce the d‐band center of the composite, which results in a relatively moderate hydrogen‐metal binding energy and increases the HER activity of the hybrid.^14^, ^19^ Furthermore, the surface oxidized Ni can accelerate water dissociation to H* (the Volmer step) and Ru can boost the combination of H* to H_2_ through efficient synergistic effect.^8^, ^20^ Therefore, the combination of Ru and Ni can not only cut down the material cost, but also significantly enhance the electrocatalytic activity caused by the shift of charge distribution and the resulting modification of surface properties.^15^, ^21^

The construction of unique nanostructure is an effective route to improve the performance of heterogeneous catalyst.^22^, ^23^ In particular, the small size and highly dispersed metal nanoparticles (NPs) have attracted considerable attention in multiphase catalysis because they can exhibit some special properties, including distinctive geometries, unique electronic characteristics, and more surface active atoms.^5^ Nevertheless, the grievous agglomeration of metal NPs with a high surface energy usually induce a severe degradation of catalytic activity and reusability.^24^, ^25^ Loading NPs onto conductive carbon matrices can effectively avoid these disadvantages and improve the electronic conductivity of composites. Simultaneously, the carbon layer can protect the internal NPs leaching into the electrolyte and avoid the corrosion and oxidation caused by the external environment, thus allowing the catalyst with high persistence. In addition, heteroatom‐doping, such as nitrogen‐doping, into carbon materials, can activate adjacent carbon atoms and modify the electronic structure of the carbon matrices, consequently, highly expand the electrochemically active surface area.^26^, ^27^ Among diverse carbon‐based carriers, 1D carbon nanofibers (CNFs) have received extensive attention in the field of electrochemical energy owing to their large specific surface area, short mass diffusion distance, and fast electron transport.^28^ However, in order to immobilize NPs on the carbon skeleton, previous studies usually involve a variety of complex synthetic processes and NPs are easily detached from the carbon carrier.^29^ Fortunately, electrospinning can firmly encapsulate NPs into CNFs in simple steps, and the metal/CNFs with a large specific surface area, small grain size, and homogeneous morphology can be prepared.^30^ Furthermore, the electrospun nanomaterials possess a broad prospect in practical application owing to the advantages of facile operation, friendly environment, low cost, and large production capacity.^31^, ^32^

In this study, a facile and cost‐effective electrospinning technique combined with carbonization process has been adopted to fabricate Ru and Ni nanoparticles‐embedded in nitrogen‐doped CNFs (RuNi‐NCNFs) as both superior HER and OER electrocatalysts. Although there have been some studies on RuNi electrocatalysts, the preparation of RuNi nanoparticles embedded in CNFs via electrospinning for water splitting has not been reported. 1D nanostructure with tunable compositions can be obtained by regulating the content of RuCl_3_ and Ni(NO_3_)_2_ in precursor solution during the electrospinning process. Compared with other synthetic approaches, the N‐doping and the incorporation of Ru/Ni catalysts can be simultaneously realized, which avoid the complicated procedure. Benefiting from the synergistic effect between Ru and Ni, conductive N‐doped carbon supports, and large active surface area, our prepared RuNi‐NCNFs catalyst exhibits excellent HER catalytic activity in both alkaline and acidic media, with low overpotentials of 35 and 23 mV (at *j* = 10 mA cm^−2^), which are comparable with the state‐of‐the‐art Pt‐based catalyst. In addition, this catalyst also exhibits an outstanding OER activity in an alkaline solution, with a low overpotential of 290 mV (at *j* = 10 mA cm^−2^). More importantly, our home‐made alkaline electrolyzer using Ru_1_Ni_1_‐NCNFs as both anodic and cathodic catalysts only requires a cell voltage of 1.564 V to drive current density of 10 mA cm^−2^ and performs robustly during continuous operation for 20 h, superior to the integrated Pt/C and RuO_2_ counterparts. This facile, rapid, and cost‐effective method provides a novel opinion to fabricate high efficiency bifunctional electrocatalyst for overall water splitting.

As displayed in **Figure** **1**a, the PAN/RuCl_3_/Ni(NO_3_)_2_ (*n*_Ru/Ni_ = 1) nanofibrous membranes have been first fabricated via electrospinning an *N*,*N*‐dimethylformamide (DMF) solution containing RuCl_3_, Ni(NO_3_)_2_, and PAN. It can be discovered that the nanofibers have a smooth surface with an average diameter of around 1 µm (Figure S1, Supporting Information). As shown in Figure S2a,d (Supporting Information), the control samples of the electrospun PAN/RuCl_3_/Ni(NO_3_)_2_ (*n*_Ru/Ni_ = 2) and PAN/RuCl_3_/Ni(NO_3_)_2_ (*n*_Ru/Ni_ = 0.5) nanofibrous membranes have also been prepared, showing similar fiber‐like morphologies and diameters. Second, these nanofibrous membranes are carbonized at 800 °C under Ar flow to produce Ru_1_Ni_1_‐NCNFs, Ru_2_Ni_1_‐NCNFs, and Ni_2_Ru_1_‐NCNFs. The fibrous structure is well maintained while the carbon matrix suffers obvious shrinkage with an average diameter of around 515 nm for Ru_1_Ni_1_‐NCNFs (Figure 1b). As can be seen from the transmission electron microscope (TEM) image (Figure 1c), Ru and Ni nanoparticles are homogeneously distributed throughout the whole NCNFs. Similar morphologies have also been observed for Ru_2_Ni_1_‐NCNFs (Figure S2b,c, Supporting Information) and Ni_2_Ru_1_‐NCNFs (Figure S2e,f, Supporting Information). As shown in Figure S3 (Supporting Information), the average diameters of Ni and Ru nanoparticles in Ru_1_Ni_1_‐NCNFs are about 16.09 nm. We have also prepared Ru‐NCNFs and Ni‐NCNFs for comparison. Figure S4 (Supporting Information) shows the morphologies of PAN/RuCl_3_ and PAN/Ni(NO_3_)_2_ fiber membranes before carbonization, showing uniform fibrous structures. After a carbonization treatment at 800 °C, the density of nanoparticles in Ru_1_Ni_1_‐NCNFs is significantly higher than that of pure Ru‐NCNFs (Figure 1d,e) and Ni‐NCNFs (Figure 1f,g). Compared with Ru‐NCNFs, the surface of Ru_1_Ni_1_‐NCNFs is much rougher, which is due to the tendency of Ni nanoparticles to form on the surface of the fiber during carbonization process, which can be confirmed by the SEM and TEM diagrams of the prepared Ni‐NCNFs (Figure 1f,g). The combination of Ru and Ni effectively avoids the tendency of Ni nanoparticles, generating a uniform Ru_1_Ni_1_‐NCNFs. In addition, there are many pores on the surface of Ru_1_Ni_1_‐NCNFs. It is due to the fact that, with an introduction of Ni(NO_3_)_2_, a large amount of gas was generated in the carbonization process, leading to the rupture of the nanofibers and the formation of the pores on the surface of nanofibers.^33^ The existence of these nanopores is beneficial to the full infiltration of electrolyte into the catalyst, providing a large quantity of catalytic sites for HER and OER and offers substantial channels for the mass transfer.^34^ Furthermore, the high surface roughness of Ru_1_Ni_1_‐NCNFs exhibits more active sites and facilitates the decreasing contact between the gas bubbles and electrode, thus leading to the fast kinetics and enhanced electrocatalytic activity. Furthermore, since the carbonization temperature is significant for the catalytic activity of carbon‐based electrocatalysts, Ru_1_Ni_1_‐NCNFs that are prepared under different carbonization temperatures have also been investigated. As shown in Figure S5a (Supporting Information), the nanofibers carbonized at 700 °C still maintain 1D morphology, but there is almost no pores on the surface of the fibers. The crystallinity of the nanoparticles is relatively inferior due to the insufficient degree of carbonization, and the morphology of the particles is not easily observed from the TEM image (Figure S5b, Supporting Information). When the temperature is raised to 900 °C, gaps and cracks appear on the surface of the fiber, and the nanoparticles tend to be agglomerated (Figure S5c,d, Supporting Information).

**Figure 1:**
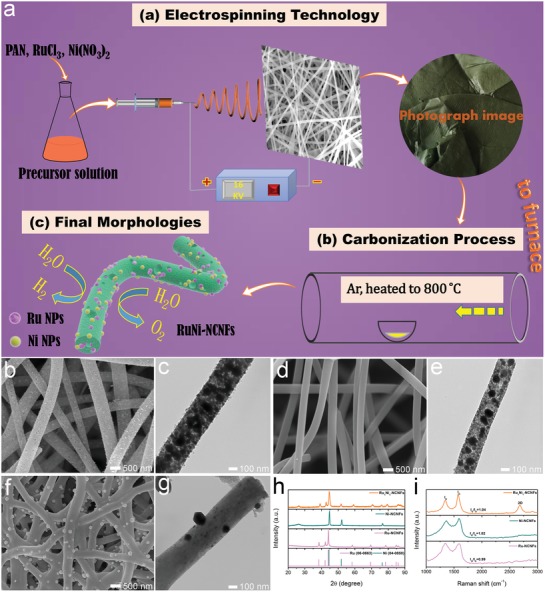
a) Schematic illustration for the fabrication process of RuNi‐NCNFs electrocatalysts. SEM images of b) Ru_1_Ni_1_‐NCNFs, d) Ru‐NCNFs, and f) Ni‐NCNFs. TEM images of c) Ru_1_Ni_1_‐NCNFs, e) Ru‐NCNFs, and g) Ni‐NCNFs. h) XRD patterns and i) Raman spectra of Ru_1_Ni_1_‐NCNFs, Ru‐NCNFs, and Ni‐NCNFs.

The X‐ray diffraction (XRD) measurement obviously identifies the harvest of Ru–Ni heterostructures in the final products (Figure S6, Supporting Information), in which the phases of Ru (JCPDS NO.: 06–0663) and Ni (JCPDS NO.: 04–0850) are clearly observed. With the enlargement of the content of Ru, the crystallization property and peak strength enhance obviously. The same phenomenon happens when the proportion of Ni increases. However, the pattern of Ru in Ru_1_Ni_1_‐NCNFs catalyst displays a slight shift to higher diffraction angle in comparison with Ru‐NCNFs and the peaks of Ni in Ru_1_Ni_1_‐NCNFs migrate to a lower angle compared to Ni‐NCNFs, revealing the strong interactions between Ru and Ni components in the Ru_1_Ni_1_‐NCNFs sample (Figure 1h). Figure S7 (Supporting Information) exhibits the XRD pattern of Ru_1_Ni_1_‐NCNFs that are carbonized at different temperatures. It can be observed that with the increment of carbonization temperature, the crystallization and peak intensity of Ru and Ni increase gradually. Figure 1i displays the Raman spectra of the prepared samples which feature three peaks at 1343 cm^−1^ for the D band (disordered carbon atoms), 1575 cm^−1^ for the G band (hybridized graphitic carbon atoms), and 2684 cm^−1^ for the 2D band (graphitic sp^2^‐bonded carbon atoms).^35^, ^36^ The ratio of intensities between the G‐band and D‐band (*I*_G_/*I*_D_) is corresponding to the degree of graphitization of carbon. It is proved that the addition of a Ni source is propitious to the formation of the highly graphitized carbon material. Thus, the maximum *I*_G_/*I*_D_ value and obvious 2D band of Ru_1_Ni_1_‐NCNFs indicate that the carbon structure produced by the synergistic effect of Ru and Ni has a higher graphitization degree, which may promote the charge transfer of the electrocatalytic process.^37^

High‐resolution TEM (HRTEM) images (**Figure** **2**a,b) display the typical spacings of 0.20 and 0.24 nm corresponding to the (111) plane of Ni and the (100) plane of Ru, respectively, demonstrating the successful formation of Ni and Ru nanoparticles. Figure 2c shows the selected area electron diffraction (SAED) pattern and the legible rings can be ascribed to the crystal planes of Ni and Ru, suggesting a polycrystalline characteristic of Ni and Ru within NCNFs. Furthermore, the energy dispersive X‐ray (EDX) presents strong Ru, Ni, C, and O signals in Ru_1_Ni_1_‐NCNFs (Figure 2d). Elemental mapping displays the uniform distribution of Ni and Ru nanoparticles in the entire NCNFs (Figure 2e). Notably, most of the Ni nanoparticles are adjacent to Ru nanoparticles, indicating possible electron transfer between them. We can also observe that C and N are distributed homogeneously among the whole nanofibers, suggesting the uniform N doping in the carbon nanofibers, which may contribute to an excellent electrocatalytic activities. According to the inductively coupled plasma atomic emission spectrometry (ICP‐AES) measurement, the weight percentages of Ni and Ru in Ru_1_Ni_1_‐NCNFs are estimated to be 16.4 and 28.2 wt%, respectively.

**Figure 2:**
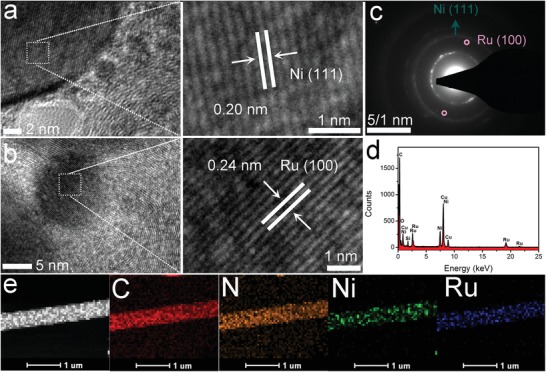
a,b) HRTEM images of Ni (a) and Ru (b). c) SAED pattern and d) EDX spectrum of the fabricated Ru_1_Ni_1_‐NCNFs. e) HAADF‐STEM image and EDX mapping of C, N, Ni, and Ru elements of Ru_1_Ni_1_‐NCNFs.

The surface compositions of the as‐prepared samples are further investigated by X‐ray photoelectron spectroscopy (XPS) to shed light on the chemical and structural information. As shown in **Figure** **3**a, the Ru 3p core spectrum of Ru_1_Ni_1_‐NCNFs exhibits a doublet situated at 462.2 and 484.5 eV, assigning to metallic Ru 3p_3/2_ and Ru 3p_1/2_ states.^38^ This result further manifests that Ru^3+^ is almost completely reduced to Ru^0^ species during carbonization process, which are highly expected for displaying satisfactory electrocatalytic activity for HER. Compared with Ru‐NCNFs, the Ru 3p peaks of Ru_1_Ni_1_‐NCNFs shift 0.2–0.3 eV in negative direction. As shown in Figure 3b, the Ni 2p_3/2_ and Ni 2p_1/2_ peaks appear at 853.8 and 870.8 eV with a splitting of 17 eV, demonstrating the formation of metallic Ni atoms in Ru_1_Ni_1_‐NCNFs. Furthermore, Ni 2p_3/2_ and Ni 2p_1/2_ signals at 856.3 and 874.4 eV with their corresponding satellite peaks at 860.8 and 880.8 eV suggest the oxidation of some surface of Ni atoms to NiO.^39^ It is worth noting that Ni^2+^ species can effectively boost water dissociation to H* and that Ru can promote the combination of H* to H_2_ through a synergistic effect.^40^, ^41^ Compared with Ni‐NCNFs, Ni 2p peaks in the XPS spectrum of Ru_1_Ni_1_‐NCNFs shift to a higher binding energy. The binding energy shifts of the Ni 2p and Ru 3p are in the opposite direction, which can be ascribed to the charge transfer from less electronegative Ni to more electronegative Ru across their interfaces.^42^ The synergistic interaction between Ru and Ni metals in Ru_1_Ni_1_‐NCNFs caused by effective electronic transfer can result in a relatively moderate hydrogen‐metal binding energy and improve the catalytic performance of HER. In order to investigate the effect of different ratios of RuCl_3_ and Ni(NO_3_)_2_ on the valence states of Ru and Ni in RuNi‐NCNFs, the electrocatalysts of Ru_2_Ni_1_‐NCNFs and Ni_2_Ru_1_‐NCNFs are also characterized by XPS measurement. As displayed in Figure S8 (Supporting Information), the samples with different Ru/Ni ratios have little effect on the valence state of metallic Ru. However, when the molar ratio of Ru to Ni is 1:2 and 2:1, the content of Ni^2+^ is higher than that of Ni^0^ species. The C1s peak of Ru_1_Ni_1_‐NCNFs can be deconvoluted into three independent peaks at 284.6, 285.2, and 280.3 eV (Figure S9a, Supporting Information), which corresponds to C=C, C—N, and the overlapped Ru 3d_5/2_ (Ru^0^), respectively.^43^, ^44^ The N 1s spectrum displays the characteristic peaks at 398.7, 401.0, and 402.5 eV, which are due to pyridinic N, pyrrolic N, and graphitic N, respectively (Figure S9b, Supporting Information).^45^, ^46^ N‐doping can effectively enhance the electrocatalytic activities in HER and OER due to their function in modulating structural and electronic changes.^47^, ^48^ As shown in Figure S9c (Supporting Information), the O 1s spectrum can be deconvoluted into two contributions centered at 532.2 and 530.0 eV, which are indexed to the spectra for the C—O and Ni—O groups stemmed from the partial oxidized surface Ni atoms, respectively.^49^

**Figure 3:**
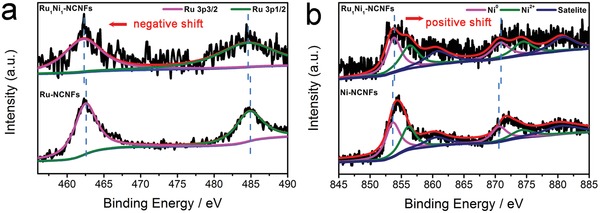
a) Ru 3p XPS spectra of Ru_1_Ni_1_‐NCNFs and Ru‐NCNFs. b) Ni 2p XPS spectra of Ru_1_Ni_1_‐NCNFs and Ni‐NCNFs.

We have employed a typical three‐electrode configuration to assess the HER performance of the as‐prepared electrocatalysts in Ar‐saturated 1 m KOH. First, the HER activity of three electrocatalysts with different molar ratios of Ru and Ni is evaluated. Figure S10a (Supporting Information) provides one cycle of the cyclic voltammetries (CVs) together with the curve after *iR‐*correction of the as‐synthesized Ru_1_Ni_1_‐NCNFs, which exhibits the lowest overpotentials of 35 and 80 mV at 10 and 50 mA cm^−2^, respectively. These values are better than those of the Ru_2_Ni_1_‐NCNFs (49 mV at 10 mA cm^−2^) and the Ni_2_Ru_1_‐NCNFs (186 mV at 10 mA cm^−2^) (Figure S11a, Supporting Information), indicating that metallic Ru is the main active source of HER. Subsequently, we have investigated the HER performances of a series of Ru_1_Ni_1_‐NCNFs obtained at different carbonization temperatures. To obtain the current density of 10 mA cm^−2^, the overpotentials required for Ru_1_Ni_1_‐NCNFs‐700 and Ru_1_Ni_1_‐NCNFs‐900 catalysts are 48 and 83 mV, respectively, which are higher than that of Ru_1_Ni_1_‐NCNFs‐800, suggesting that 800 °C is the optimal carbonization temperature (Figure S12a, Supporting Information). Ru‐NCNFs, Ni‐NCNFs, and commercial Pt/C (20 wt%) are also tested under the same conditions (**Figure** **4**a). Strikingly, the catalyst of the Ru_1_Ni_1_‐NCNFs exhibits the best electrocatalytic performance with a near zero onset overpotential. The overpotential required for Ru_1_Ni_1_‐NCNFs to reach 10 mA cm^−2^ is lower than that of Ru‐NCNFs (45 mV), Ni‐NCNFs (233 mV), and the state‐of‐the‐art Pt/C (37 mV). Due to the excellent intrinsic activity of metallic Ru, Ru‐NCNFs perform much better HER activity than Ni‐NCNFs. Notably, Ru_1_Ni_1_‐NCNFs display much lower overpotential values than Pt/C at *j*_HER_ > 8.4 mA cm^−2^, indicating that Ru_1_Ni_1_‐NCNFs exhibit better HER performance than commercial Pt/C at high current densities. For the physical mixture of Ru‐NCNFs and Ni‐NCNFs, the HER activity is located between those of its counterpart catalysts and is considerably inferior to that of the Ru_1_Ni_1_‐NCNFs catalyst. It is proved that the synergistic effect of chemical coupling of Ru and Ni metals by electrospinning technique is the main reason to enhance the performance of HER. As displayed in Figure 4b, Ru_1_Ni_1_‐NCNFs reveal a Tafel slope of 30 mV dec^−1^, which is much lower than those of Ru‐NCNFs (35 mV dec^−1^), Ni‐NCNFs (79 mV dec^−1^), and the physical mixture of Ru‐NCNFs and Ni‐NCNFs (41 mV dec^−1^) and identical with that of Pt/C (30 mV dec^−1^), demonstrating a faster HER kinetics of Ru_1_Ni_1_‐NCNFs. In light of the Tafel slope range, the HER catalyzed by Ru_1_Ni_1_‐NCNFs in this system is presumably conducted by a Volmer–Tafel mechanism. The inherent HER activity of these catalysts has been evaluated by the exchange current density (*j*_0_) according to the Tafel plots. Remarkably, the Ru_1_Ni_1_‐NCNFs catalyst exhibits the largest *j*_0_ of 0.745 mA cm^−2^ among the as‐prepared catalysts and is nearly close to that of Pt/C (0.920 mA cm^−2^). It is commonly known that a large j_0_ is generally associated with the large electrochemically active surface area (ECSA),^50^ which can be determined by gauging the double layer capacitance (*C*_dl_) through CV measurements at various scan rates (Figure S13, Supporting Information).^51^ The *C*_dl_ of 44.27 mF cm^−2^ for Ru_1_Ni_1_‐NCNFs is larger than the 34.1 mF cm^−2^ for Ru‐NCNFs and 26.99 mF cm^−2^ for Ni‐NCNFs, revealing that more active sites are available for the Ru_1_Ni_1_‐NCNFs catalyst (Figure 4c). Furthermore, the ECSA of the different catalyst can be obtained in terms of the equation: ECSA = *C*_dl_/*C*_s_, where *C*_s_ is the specific capacitance for a flat surface (1 cm^2^). Then the ECSA values are proportional to the *C*_dl_ values according to typical reported values.^52^, ^53^ Therefore, the Ru_1_Ni_1_‐NCNFs possess the largest ECSA compared with the Ru‐NCNFs and Ni‐NCNFs. The large *j*_0_ and high ECSA indicate that the Ru_1_Ni_1_‐NCNFs catalyst with high surface roughness reveals more active sites and promotes the rapid formation and separation of hydrogen bubbles on the electrode surface, giving rise to the fast kinetics and increased HER activity.^50^ In order to better understand the intrinsic activity, the turnover frequency (TOF) of HER in an alkaline solution has been estimated. The Ru_1_N_1_‐NCNFs catalyst possesses a high TOF of 0.076 s^−1^ at overpotential of 80 mV, which is larger than most of recently reported catalysts (Table S1, Supporting Information). The high TOF of Ru_1_Ni_1_‐NCNFs is agreement with its outstanding activity toward the HER. The mass activity (MA) has also been calculated to further evaluate the intrinsic activity. It is found that the MA value of Ru_1_Ni_1_‐NCNFs (16.44 A g^−1^) is the highest among the all samples (Figure S14a, Supporting Information). Furthermore, the advantageous electrical conductivity of Ru_1_Ni_1_‐NCNFs enables rapid electron transport, which was confirmed by the electrochemical impedance spectroscopy (EIS). As the Nyquist diagram shown in Figure 4d, the Ru_1_Ni_1_‐NCNFs manifest a charge‐transfer resistance (*R*_ct_) of 9.11 Ω, which is much lower than that of pure Ru‐NCNFs or Ni‐NCNFs. The smallest *R*_ct_ of the Ru_1_Ni_1_‐NCNFs catalyst can be ascribed to its porous composite structure, which enables ultrafast faradaic processes and superior HER kinetics. To evaluate the durability of Ru_1_Ni_1_‐NCNFs, continuous CV cycles were carried out on the rotating disk electrode (RDE) at a scan rate of 100 mV s^−1^. As shown in Figure 4e, after 5500 CV cycles, the curves present negligible changes, demonstrating that the Ru_1_Ni_1_‐NCNFs catalyst displays excellent stability for HER. The chronoamperometric test was conducted under a constant overpotential of 62 mV for Ru_1_Ni_1_‐NCNFs on carbon paper with a loading of 2.5 mg cm^−2^ without *iR*‐compensation and the corresponding polarization plot is shown in Figure S15a (Supporting Information). After a continuous 12 h electrolysis, it displays superior durability with merely very little decline (Figure 4f). Therefore, the HER performance of this novel Ru_1_Ni_1_‐NCNFs catalyst is far superior to most previously documented electrocatalysts in alkaline media (Table S2, Supporting Information).

**Figure 4:**
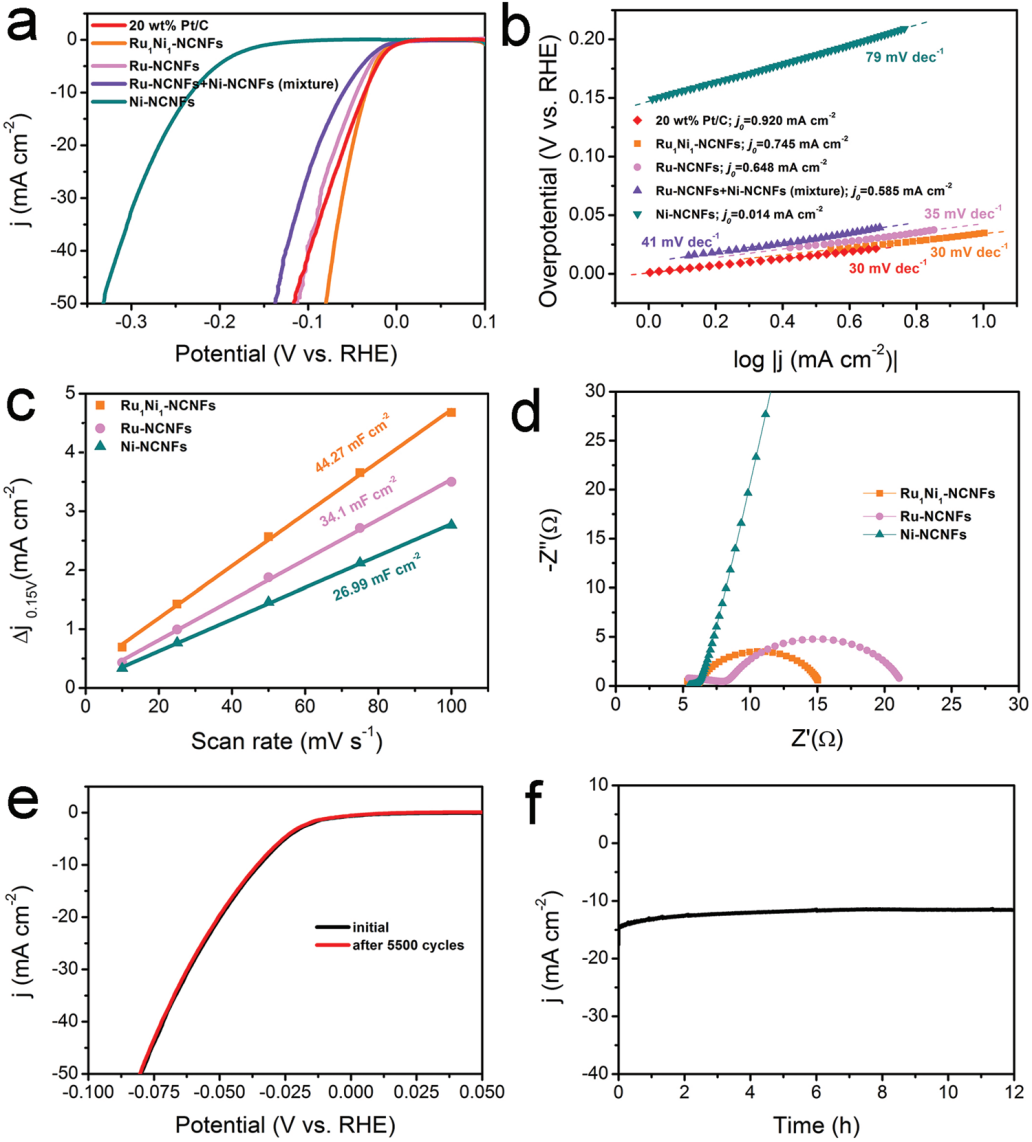
HER tests in 1 m KOH. a) The HER polarization curves for as‐prepared catalysts at a scan rate of 1 mV s^−1^. b) Tafel plots and exchange currents for different catalysts. c) The relationship between capacitance current and scanning rate at 0.15 V potential. The double‐layer capacitance (*C*_dl_) of the catalyst is the slope after the linear fit. d) Nyquist diagram (overpotential = 60 mV) for different samples. e) Polarization curves before and after 5500 CV cycles. The mass loading is 0.612 mg cm^−2^ loaded on RDE for the measurements in (a–e). f) The chronoamperometric test (without *iR*‐drop compensation) was conducted under a constant overpotential of 62 mV for Ru_1_Ni_1_‐NCNFs on carbon paper with a loading of 2.5 mg cm^−2^.

The HER catalytic activity in acidic media has been further investigated. The CV curves of the Ru_1_Ni_1_‐NCNFs without or with *iR‐*compensation in 0.5 m H_2_SO_4_ at a scan rate of 1 mV s^−1^ are exhibited in Figure S10c (Supporting Information). As shown in Figures S11c and S12c (Supporting Information), when the molar ratio of Ru to Ni is 1:1 and the carbonization temperature is 800 °C, the best HER performance of RuNi‐NCNFs in an acidic electrolyte is achieved. The polarization plots of different samples in 0.5 m H_2_SO_4_ are displayed in Figure S16a (Supporting Information). It is clearly remarkable that the onset overpotential of Ru_1_Ni_1_‐NCNFs is about 0 mV, and the overpotential at the current density of 10 mA cm^−2^ is 23 mV, which is comparable to the commercial Pt/C (17 mV). The η_10_ of the physical mixture (63 mV) is between the value of Ru‐NCNFs (50 mV) and Ni‐NCNFs (389 mV) and is extremely higher than the Ru_1_Ni_1_‐NCNFs, further confirming that the chemical coupling between Ru and Ni produces catalytically synergistic effect. The Tafel slope for Ru_1_Ni_1_‐NCNFs is 29 mV dec^−1^, indicating that a Volmer–Tafel reaction mechanism controlled the HER process and that electrochemical desorption is the rate‐determining step (H* + H^+^ + e ^−^→ H_2_) in acidic media (Figure S16b, Supporting Information).^54^ The MA value of Ru_1_Ni_1_‐NCNFs (16.83 A g^−1^) was ≈4.4‐times and 12‐folds than Ru‐NCNFs and Ni‐NCNFs, respectively (Figure S14c, Supporting Information). The Ru_1_Ni_1_‐NCNFs catalyst reveals a high TOF of 0.074 s^−1^ at an overpotential of 55 mV, which is superior to other recently reported HER catalysts (Table S3, Supporting Information), indicating that Ru_1_Ni_1_‐NCNFs possess a much higher intrinsic activity for the HER in an acidic condition. Furthermore, we also estimated the electrocatalytic HER stability in 0.5 m H_2_SO_4_. Figure S16c,d (Supporting Information) displays the linear sweep voltammetry (LSV) curves of Ru_1_Ni_1_‐NCNFs before and after 6000 CV cycles and the *i*–*t* curve at an overpotential of 40 mV without *iR*‐compensation, respectively. After the long‐term durability measurements, no evident variations are observed in both polarization and *i*–*t* curves, proving Ru_1_Ni_1_‐NCNFs with outstanding HER stability in acidic electrolyte. Therefore, our‐prepared Ru_1_Ni_1_‐NCNFs catalyst outperforms most of the recently developed HER electrocatalysts in acidic media (Table S4, Supporting Information).

Furthermore, the Ru_1_Ni_1_‐NCNFs catalyst was also observed to be active for OER in 1 m KOH. Figure S10b (Supporting Information) provides the CV curves before and after *iR*‐compensation of the as‐synthesized sample. The OER activities of RuNi‐NCNFs with three different molar ratios of Ru and Ni have been first evaluated, and the results are shown in Figure S11b (Supporting Information). It is very clear that the Ru_1_Ni_1_‐NCNFs display a low overpotential of 290 mV with respect to Ni_2_Ru_1_‐NCNFs (320 mV) and Ru_2_Ni_1_‐NCNFs (380 mV) to reach a current density of 10 mA cm^−2^. These results indicate that the optimum ratio between Ru and Ni is 1:1, and metallic Ni is the main active component for OER. Afterward, we have optimized the carbonization temperature of Ru_1_Ni_1_‐NCNFs. As shown in Figure S12b (Supporting Information), the overpotentials required by Ru_1_Ni_1_‐NCNFs‐700 and Ru_1_Ni_1_‐NCNFs‐900 to drive the current density of 10 mA cm^−2^ are 406 and 327 mV, respectively, which are higher than that required by Ru_1_Ni_1_‐NCNFs‐800, revealing that the optimum carbonization temperature of Ru_1_Ni_1_‐NCNFs is 800 °C. **Figure** **5**a displays the polarization curves of as‐prepared samples with *iR*‐drop corrections, along with that of the benchmark RuO_2_ catalyst for reference. The Ru_1_Ni_1_‐NCNFs afford a η_10_ of 290 mV, which was 50 and 143 mV less than those of the Ni‐NCNFs and the state‐of‐the‐art RuO_2_, respectively. For the physical mixture of Ni‐NCNFs and Ru‐NCNFs, its OER activity is located between those of its counterpart catalysts and is substantially inferior to that of the Ru_1_Ni_1_‐NCNFs catalyst, indicating that the strong chemical coupling effect plays a vital role in the Ru_1_Ni_1_‐NCNFs composite catalyst. The individual Ru‐NCNFs catalyst exhibits poor OER activity, and even at 1.75 V (vs reversible hydrogen electrode, RHE) voltage, its current density does not reach 10 mA cm^−2^, indicating that Ni nanoparticles in Ru_1_Ni_1_‐NCNFs are the main active species for electrocatalytic OER. In addition, from the XPS spectra (Figure 3b), we can observe that Ru_1_Ni_1_‐NCNFs possess a higher content of Ni^2+^ than Ni‐NCNFs, which may be caused by the strong electron transfer from metallic Ni to Ru, and Ni^2+^ is considered to be the active site for OER.^40^ Therefore, the enhanced OER activity of Ru_1_Ni_1_‐NCNFs derives from the synergistic effect between metal Ru and Ni. The TOF of Ru_1_Ni_1_‐NCNFs is 0.038 s^−1^ at an overpotential of 335 mV, which is much higher than those of previously reported OER electrocatalysts (Table S5, Supporting Information). Similarly, compared with other samples, the Ru_1_N_1_‐NCNFs possess the maximum MA value of 16.64 A g^−1^ at definite overpotential of 290 mV (Figure S14b, Supporting Information), confirming the excellent intrinsic electrocatalytic activity of Ru_1_Ni_1_‐NCNFs for OER. Moreover, this composite material is considerably stable, in 4500 cycles of CV test, the curve before and after the cycle almost coincides (Figure 5b). In the chronoamperometric measurement (without *iR*‐compensation), only a small decrease of the current density was observed after continuous electrolysis for 12 h (inset in Figure 5b). Although Ru and Ni are partially oxidized during the OER process, the generated Ni^3+^ helps to form NiOOH on the surface of the catalyst, resulting in a better OER performance.^14^ In addition, the formation of Ru^4+^ during the OER process is the active site for promoting OER.^55^ Therefore, both Ru and Ni in high oxidation states generated during the OER process are favorable for the enhanced OER performance. The remarkable OER performance of Ru_1_Ni_1_‐NCNFs is superior to other recently reported OER electrocatalysts in alkaline media (Table S6, Supporting Information).

**Figure 5:**
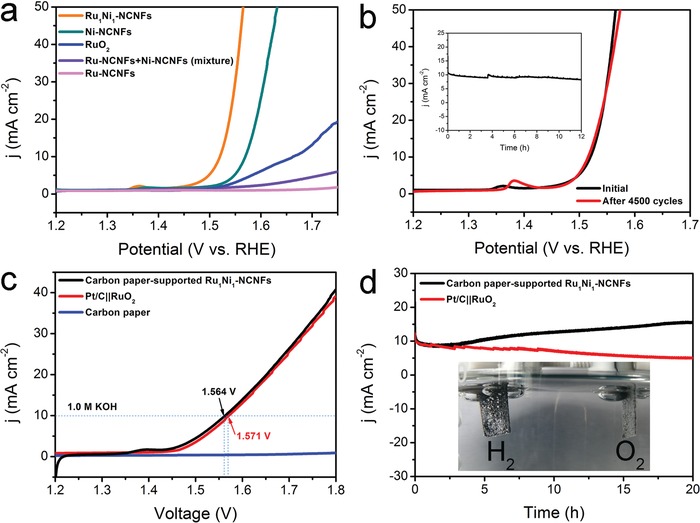
OER and water‐splitting tests in 1 m KOH. a) The OER polarization curves (with *iR*‐correction) with a mass loading of 0.612 mg cm^−2^ loaded on an RDE electrode. b) Polarization curves (with *iR*‐correction) for Ru_1_Ni_1_‐NCNFs on an RDE electrode before and after 4500 CV cycles. The inset shows the chronoamperometric test (without *iR*‐correction) that was conducted under a constant overpotential of 300 mV for Ru_1_Ni_1_‐NCNFs on carbon paper with a loading of 2.5 mg cm^−2^. c) LSV plots (without *iR*‐correction) of overall water splitting in a two‐electrode system. d) The stability (without *iR*‐correction) tests of overall water splitting for Ru_1_Ni_1_‐NCNFs and benchmark electrodes of Pt/C||RuO_2_ were all carried out at a constant voltage of 1.57 V. The loading of both cathode and anode for water splitting is 2.5 mg cm^−2^ loaded on carbon paper. The inset is the optical photograph displaying the production of H_2_ and O_2_ bubbles on the Ru_1_Ni_1_‐NCNFs electrodes.

In view of the outstanding activity and stability of Ru_1_Ni_1_‐NCNFs for both HER and OER, we further evaluated the overall water splitting activity in 1 m KOH electrolyte by adopting two‐electrode configurations. The Ru_1_Ni_1_‐NCNFs catalyst was supported on the carbon paper and was employed as both the cathode and the anode for a home‐made electrolyzer. As depicted in Figure 5c, Ru_1_Ni_1_‐NCNFs electrodes achieve a current density of 10 mA cm^−2^ at a cell voltage of 1.564 V (vs RHE) (without *iR*‐compensation) and a great deal of H_2_ and O_2_ bubbles were observed on the surface of both electrodes during electrolysis (inset in Figure 5d). The two‐electrode Faraday efficiency (Figure S17, Supporting Information) has been evaluated via water drainage. The practical H_2_ amount is well with theoretical amount. The actual yield of O_2_ is slightly lower than the theoretical value, which is attributed to the high solubility of oxygen in water and the oxidation of metallic Ru to high valence state during the early stage of electrolysis. The oxidation peak located at ≈1.40 V (vs RHE) corresponds to the oxidation of M(II) to M(III or IV) (M = Ni and Co), which is common during the electrochemical process of Ni‐based electrocatalysts.^56^ Moreover, the overall water splitting activity of the Ru_1_Ni_1_‐NCNFs stays ahead of commercial catalysts (Pt/C||RuO_2_ is 1.571 V (vs RHE)@10 mA cm^−2^) and many of the previously reported bifunctional electrocatalysts (Table S7, Supporting Information). The long‐term stability of the water‐splitting reaction was estimated by continuous operation at 1.57 V (vs RHE) (without *iR*‐compensation). There was only a little degradation at the initial time, and after that the activity remains stable or even increases, displaying the favorable chemical stability and its excellent potential for practical electrolytic water system. By contrast, the benchmark electrodes of Pt/C||RuO_2_ deliver an obvious decline of current density after 20 h, revealing their instability.

The Ru_1_Ni_1_‐NCNFs catalyst was characterized in detail after HER and OER stability tests to further prove its favorable morphology and structural stability. From the SEM and TEM images (Figure S18a,c,e, Supporting Information), it can be observed that our‐prepared Ru_1_Ni_1_‐NCNFs catalyst maintained a well fibrous morphology either after HER tests in alkaline or acidic media or after OER test in alkaline electrolyte. The nanoparticles are still firmly embedded in the carbon skeleton because carbon fibers protect the inner nanoparticles from corrosion and oxidation under strong acid and alkaline conditions.^57^ According to the corresponding EDX spectra (Figure S18b,d,f, Supporting Information), the signal peaks of Ru, Ni, C, N, and O can be detected after all stability tests. However, after HER stability test in 0.5 m H_2_SO_4_ (Figure S18f, Supporting Information), the signal intensity of Ni element is obviously weakened, which is due to the metallic Ni easily dissolved in acidic electrolyte, consistent with the results of following XRD analysis (Figure S19, Supporting Information). The Ru and Ni structure of the nanoparticles is well‐preserved after the stability tests, while in the XRD pattern after HER test in 0.5 m H_2_SO_4_, the peak intensities of metallic Ni at 51.6° and 76.0° are significantly weakened, indicating that the main active substance for catalyzing HER is metallic Ru, while the active substance for OER is metallic Ni, and the chemical coupling of Ru and Ni in carbon matrix imparts excellent bifunctional electrocatalytic activity to Ru_1_Ni_1_‐NCNFs. XPS has been further performed on Ru_1_Ni_1_‐NCNFs after stability measurements. The high‐resolution XPS spectra of Ru 3p (Figure S20a, Supporting Information) indicate that the post‐HER samples share similar features with the fresh sample, whether in 1 m KOH or 0.5 m H_2_SO_4_, whereas the post‐OER sample displays visible Ru^4+^ signals, revealing that part of Ru is converted to RuO_2_ in the electrocatalytic process, thus further improving the OER activity.^14^, ^58^ In the case of Ni 2p spectrum (Figure S20b, Supporting Information), compared with the fresh sample, the signal intensity of Ni^0^ decreases significantly and that of Ni^2+^ increases obviously after HER test in 1 m KOH. It is noteworthy that Ni^2+^ species as water dissociation promoters can crack HO—H bonds and generate H*, which is considered to be effective active sites for water dissociation (Volmer step), thereby accelerating the overall reaction rate of HER.^40^, ^41^ The signals of Ni^3+^ species appear clearly in the XPS spectrum of the post‐OER sample, which is known to be highly active for OER.^59^ Above results suggest that both Ru and Ni with high oxidation states are formed in the Ru_1_Ni_1_‐NCNFs after OER test, which are conducive to the enhanced OER activity. By observing the sample after HER test in acidic media, it is found that the signal of Ni^0^ almost disappears, whereas the majority of surface Ni in Ru_1_Ni_1_‐NCNFs is oxidized with a +2 oxidation state, which is consistent with the previous results of EDX and XRD. According to the aforementioned analysis, our prepared Ru_1_Ni_1_‐NCNFs electrocatalyst exhibits satisfactory stability and is capable to withstand continuous operation in practical applications.

The outstanding HER and OER bifunctional electrocatalytic performance of Ru_1_Ni_1_‐NCNFs is presumably ascribed to the following reasons. First, the introduction of Ni largely downshifts the d‐band center of the Ru–Ni hybrids and effectively modulates the surface environment for HER. Simultaneously, the Ru in Ru_1_Ni_1_‐NCNFs will lightly transfer electrons between the catalysis substrate and intermediate molecules and promote O—O bond generation.^14^ Furthermore, Ni^2+^ species on the surface of Ru_1_Ni_1_‐NCNFs can boost water dissociation to H* and that Ru promotes the combination of H* to H_2_ via a greatly efficient synergistic catalysis, resulting in fast kinetics of HER. In addition, the abundant Ni^3+^ can facilitate the formation of surface‐hydrated oxides and (oxy)hydroxides phases (e.g., Ni(OH)_2_ and NiOOH), which provides the main reactive sites for OER. On the other hand, nitrogen‐doped carbon nanofibers with porous surfaces improve the conductivity of the catalyst, accelerate rapid electron and mass transport, and avoid the agglomeration of nanoparticles. Heteroatom N (such as graphitic N and pyrrolic N) can affect the charge delocalization of C, thereby increasing the conductivity of materials.^60^ The presence of pyridinic N atoms at the edge of carbon reduces the barrier for the adsorption of reactants on adjacent carbon atoms, which is beneficial to the rate‐limiting first‐electron transfer. Moreover, the local electron pair of pyridine‐N group can also easily interact with oxygen/proton, thus resulting in an higher OER performance. The generation of RuO_2_ in the reaction process is also the key factor to enhance the performance of OER. Finally, the Ru_1_Ni_1_‐NCNFs with large electrochemical active surface area and surface roughness can expose more active sites, thus improving the performance of electrocatalytic reaction.

In summary, we have developed a facile and convenient strategy to fabricate Ru_1_Ni_1_‐NCNFs via electrospinning, followed by a carbonization process. The synthesized Ru_1_Ni_1_‐NCNFs catalyst possesses high conductivity, large electrochemical active surface area, and abundant active sites, thus resulting in superior activity, fast charge transport, rapid release of generated gas bubbles, and favorable durability for electrocatalytic processes. Accordingly, the robust Ru_1_Ni_1_‐NCNFs as novel bifunctional electrocatalysts revealed excellent HER and OER catalytic performance in alkaline solution. In regard to overall water splitting, a low cell potential of merely 1.564 V was required to achieve a current density of 10 mA cm^−2^, which is superior to the state‐of‐the‐art catalysts Pt/C||RuO_2_ (1.571 V@10 mA cm^−2^). Moreover, the Ru_1_Ni_1_‐NCNFs catalyst also exhibited remarkable HER performance in acidic media and the overpotential required by the Ru_1_Ni_1_‐NCNFs to drive a current density of 10 mA cm^−2^ was comparable with that of commercial Pt/C. This work hints at the opportunity to combine cheap transition metals with relatively low‐price noble metals (such as Ru), both maintaining the excellent intrinsic activity of the precious metal and reducing the cost of electrocatalyst, which is highly desirable for sustainable energy development.

## Experimental Section

*Chemicals and Reagents*: Polyacrylonitrile (PAN, *M*_w_ = 150 000 g mol^−1^), ruthenium (III) chloride hydrate (RuCl_3_·*x*H_2_O), and ruthenium(IV) oxide (RuO_2_) were commercially available from Sigma‐Aldrich. Nickel (II) nitrate hexahydrate (Ni(NO_3_)_2_·6H_2_O) and DMF were purchased from Beijing Chemical Works. Commercial 20 wt% Pt/C was provided by Johnson Matthey.

*Preparation of RuNi‐NCNFs*: Typically, 0.5 g of PAN was added into 4.5 g of DMF and stirred at 90 °C for 1 h. 0.244 g of Ni(NO_3_)_2_·6H_2_O and 0.174 g of RuCl_3_·*x*H_2_O were then added into the PAN solution (the molar ratio of Ru to Ni was 1:1) and stirred vigorously until to yield a homogeneous solution. Subsequently, the precursor solution was inhaled into a syringe with a single stainless steel nozzle for electrospinning. The power supply was maintained at 16 kV between the spinneret and the aluminum foil collector with a distance of 24 cm. In the following, the as‐collected PAN/RuCl_3_/Ni(NO_3_)_2_ fiber membrane was heated at 280 °C for 2 h in air atmosphere, followed by carbonization at 800 °C for 5 h in argon to prepare Ru and Ni nanoparticles encapsulated in N‐doped CNFs (denoted as Ru_1_Ni_1_‐NCNFs). In addition, the RuNi‐NCNF electrocatalysts with molar ratios of Ru to Ni = 2:1 and 1:2 were labeled as Ru_2_Ni_1_‐NCNFs and Ni_2_Ru_1_‐NCNFs (the total moles of Ru and Ni are fixed as 1.68 mmol). The pure Ru‐NCNFs and Ni‐NCNFs were fabricated similarly with that of Ru_1_Ni_1_‐NCNFs, only without the addition of Ni(NO_3_)_2_·6H_2_O and RuCl_3_·*x*H_2_O, respectively. For comparison, the electrospun PAN/RuCl_3_/Ni(NO_3_)_2_ (*n*_Ru/Ni_ = 1) fiber membrane carbonized at 700 and 900 °C (expressed as Ru_1_Ni_1_‐NCNFs‐700 and Ru_1_Ni_1_‐NCNFs‐900) were fabricated with the other conditions unchanged.

*Characterization*: The morphology and structure of the resulting products were studied using a scanning electron microscope (FEI Nova NanoSEM) and a TEM (JEOL JEM‐2000 EX). HRTEM images and EDX spectrum were obtained using an FEI Tecnai G2 F20. The crystallographic structures of the samples were characterized by XRD (PANalytical B.V. Empyrean) with Cu Kα radiation. Raman tests were carried out on a Horiba LabRAM HR Evolution apparatus. XPS (Thermo Scientific ESCALAB250) was used to analyze the surface elemental composition of the synthesized materials. ICP‐AES (Agilent 725) was employed to estimate the weight percentages of Ni and Ru in the Ru_1_Ni_1_‐NCNFs.

*Electrochemical Measurements*: All electrochemical measurements were conducted on a CHI760E electrochemical workstation in a three electrode setup, in which a RDE made of glassy carbon electrode (geometric area: 0.196 cm^2^) with catalysts was used as the working electrode, a standard Hg/HgO electrodes as the reference (in 1 m KOH), a graphite rod and a Pt wire as the counter electrodes for HER and OER, respectively. In 0.5 m H_2_SO_4_, RHE is directly used as reference electrode. The calibration of the Hg/HgO was performed in a conventional three‐electrode system with platinum plates as the working electrode and counter electrode and the Hg/HgO as a reference electrode. The current–voltage scans were measured in high purity hydrogen (H_2_) saturated 1 m KOH solution at a scan rate of 1 mV s^−1^, and the average of the two potentials at which the current crossed zero was recorded to be the thermodynamic potential for the hydrogen electrode reaction (Figure S21, Supporting Information). The zero current potential was −0.93 V, so *E* (RHE) = *E* (Hg/HgO) + 0.93 V. The catalyst ink was prepared by putting samples (2 mg) to disperse in ethanol (500 µL), and then the mixture was sonicated for at least 30 min to form a homogeneous solution. After that, 30 µL of the dispersion was casted onto the RDE, followed by the coverage of 5 µL 0.5 wt% Nafion solution with a catalyst loading of about 0.612 mg cm^−2^. The commercial Pt/C and RuO_2_ modified electrodes were also made in the same way for comparison. LSV was conducted at a scan rate of 1 mV s^−1^ with a rotation rate of 1600 rpm. The computation of TOF value is according to the equation listed below: TOF_HER_ = *j* ×  S/(2*Fn*) and TOF_OER_ = *j* ×  S/(4*Fn*). 61 Here, *j* represents the current density (A cm^−2^) at a given overpotential, *S* is the value of the surface area for RDE electrode (0.196 cm^2^). The constants 4 and 2 are the number of electrons required to product each mole of O_2_ and H_2_, respectively. *F* is the value of the Faraday constant (96 485.3 C mol^−1^), and *n* (mol) represents the moles of the metal atoms on the electrode calculated from ICP results of the Ru_1_Ni_1_‐NCNFs. It is assumed that all Ru and Ni atoms are active and contribute to the catalytic reaction (the minimum values of TOF are calculated). The mass activity (MA, A g^−1^) values were compulated at a definite overpotential by following equation: MA =$\frac{j}{M},$where *j* (mA cm_geo_^−2^) is measured current density; *M* (mg cm_geo_^−2^) is mass loading of the catalysts.^62^ EIS was carried out at an AC‐voltage amplitude of 5 mV, for frequency ranging from 10^6^ to 0.1 Hz.

The catalyst was supported on carbon paper with a mass loading of 2.5 mg cm^−2^ for HER and OER chronoamperometric tests in a three‐electrode system. The Ru_1_Ni_1_‐NCNFs catalyst anchored on carbon paper was also used as both cathode and anode in a two‐electrode configuration for overall water splitting, and the polarization curve was measured at a scan rate of 1 mV s^−1^.

## Conflict of Interest

The authors declare no conflict of interest.

## Supporting information

Supporting Information
