# Supplementary material for: RuNi Nanoparticles Embedded in N‐Doped Carbon Nanofibers as a Robust Bifunctional Catalyst for Efficient Overall Water Splitting
Source: Adv Sci (Weinh). 2019 Nov 18;7(2):1901833. doi: 10.1002/advs.201901833 (PMC6974957; doi:10.1002/advs.201901833)
Supplement: Supplementary file 1 — Supporting Information [file ADVS-7-1901833-s001.pdf]

Copyright WILEY-VCH Verlag GmbH & Co. KGaA, 69469 Weinheim, Germany, 2019.

ADVANCED SCIENCE
Open Access

Supporting Information

for *Adv. Sci.,* DOI: 10.1002/advs.201901833

RuNi Nanoparticles Embedded in N-Doped Carbon Nanofibers as a Robust Bifunctional Catalyst for Efficient Overall Water Splitting

*Meixuan Li, Huiyuan Wang, Wendong Zhu, Weimo Li, Ce Wang, and Xiaofeng Lu**

Copyright WILEY-VCH Verlag GmbH & Co. KGaA, 69469 Weinheim, Germany, 2019.

Supporting Information

RuNi Nanoparticles Embedded in N-Doped Carbon Nanofibers as a Robust Bifunctional Catalyst for Efficient Overall Water Splitting

*Meixuan Li, Huiyuan Wang, Wendong Zhu, Weimo Li, Ce Wang and Xiaofeng Lu**

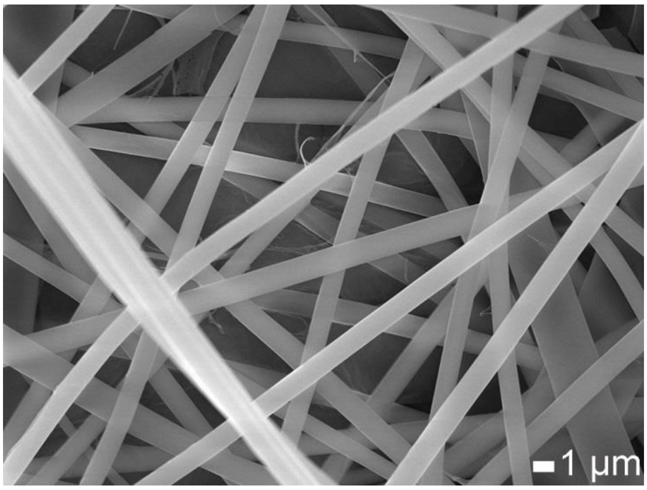

Figure S1. SEM image of the electrospun PAN/$RuCl_3$/$Ni(NO_3)_2$ ($n_{Ru/Ni}$=1) nanofibers.

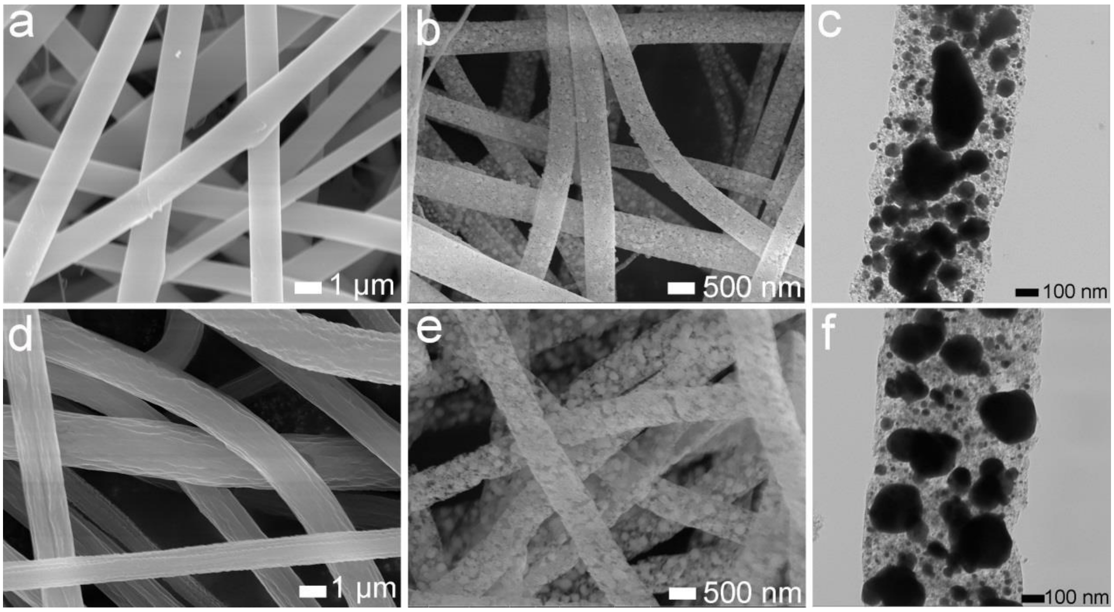

Figure S2. SEM images of the electrospun (a) PAN/$RuCl_3$/$Ni(NO_3)_2$ ($n_{Ru/Ni}$=2) nanofibers and (d) PAN/$RuCl_3$/$Ni(NO_3)_2$ ($n_{Ru/Ni}$=0.5) nanofibers. SEM images of (b) Ru_2Ni_1-NCNFs and (e) Ni_2Ru_1-NCNFs. TEM images of (c) Ru_2Ni_1-NCNFs and (f) Ni_2Ru_1-NCNFs.

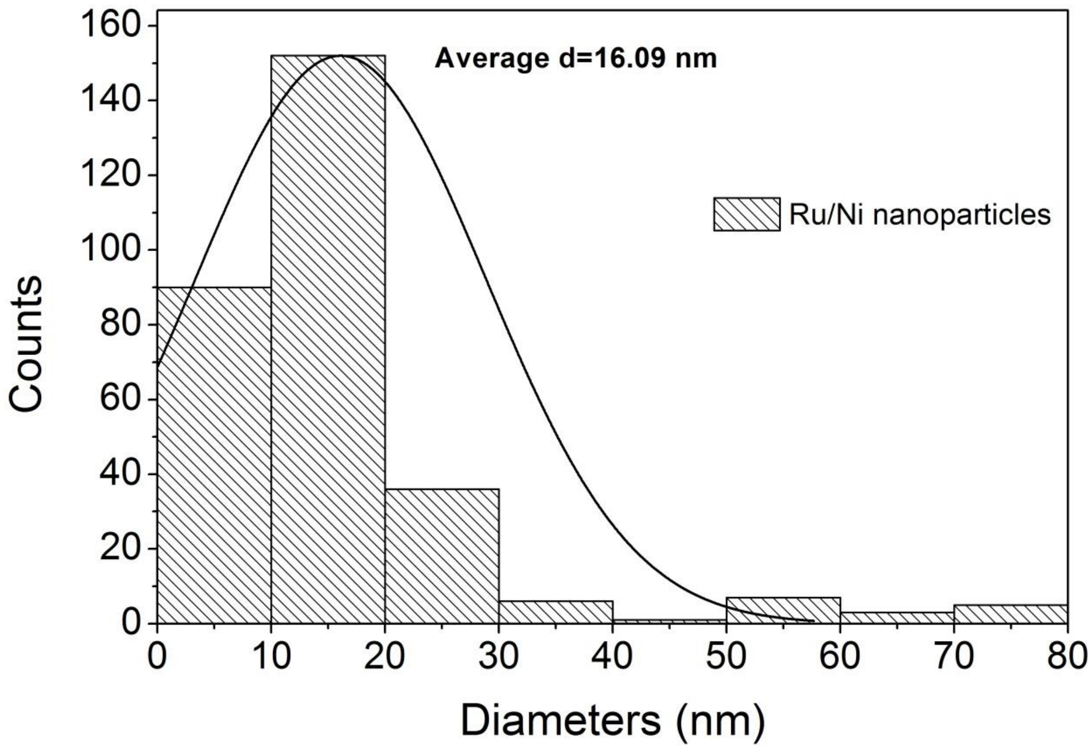

Figure S3. The diameter distributions of Ni and Ru nanoparticles in Ru_1Ni_1-NCNFs.

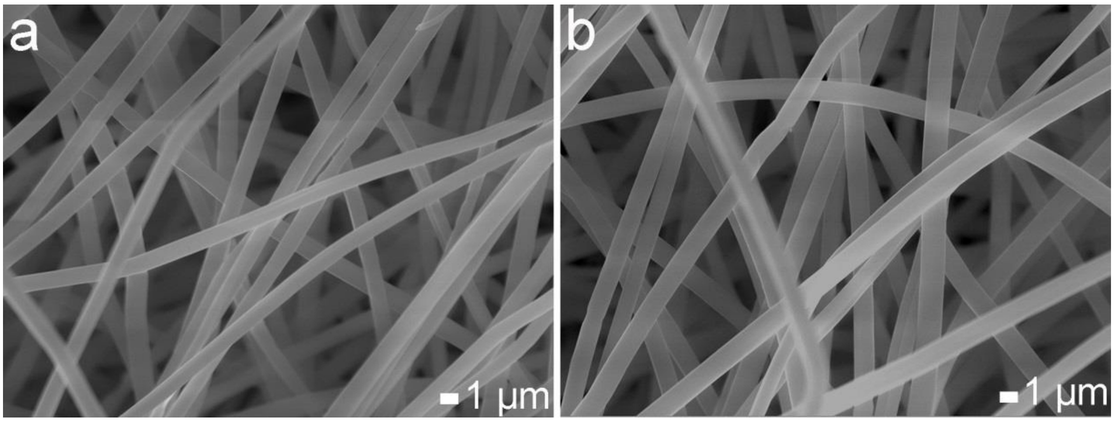

Figure S4. SEM images of the electrospun (a) PAN/$RuCl_3$ nanofibers and (b) PAN/$Ni(NO_3)_2$ nanofibers .

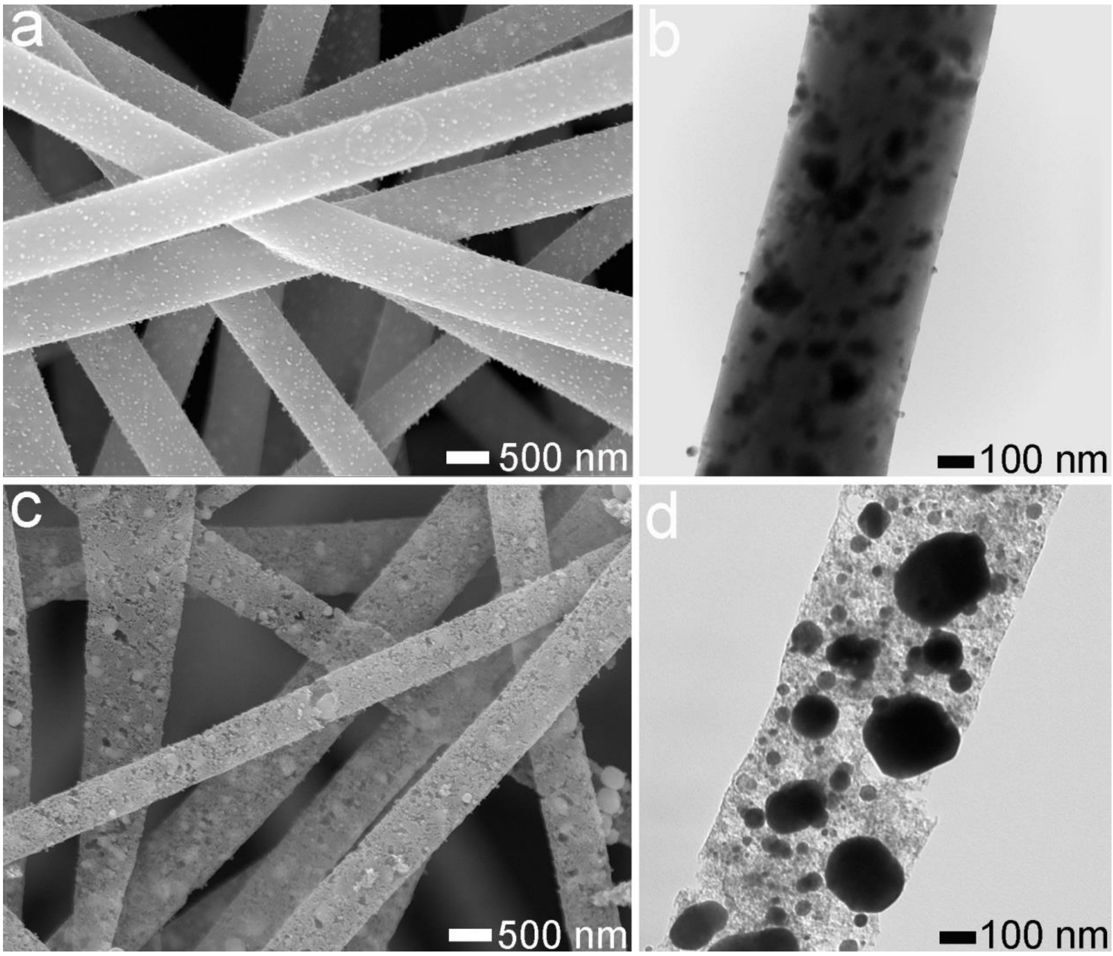

Figure S5. SEM images of (a) Ru_1Ni_1-NCNFs-700 and (c) Ru_1Ni_1-NCNFs-900. TEM images of (b) Ru_1Ni_1-NCNFs-700 and (d) Ru_1Ni_1-NCNFs-900.

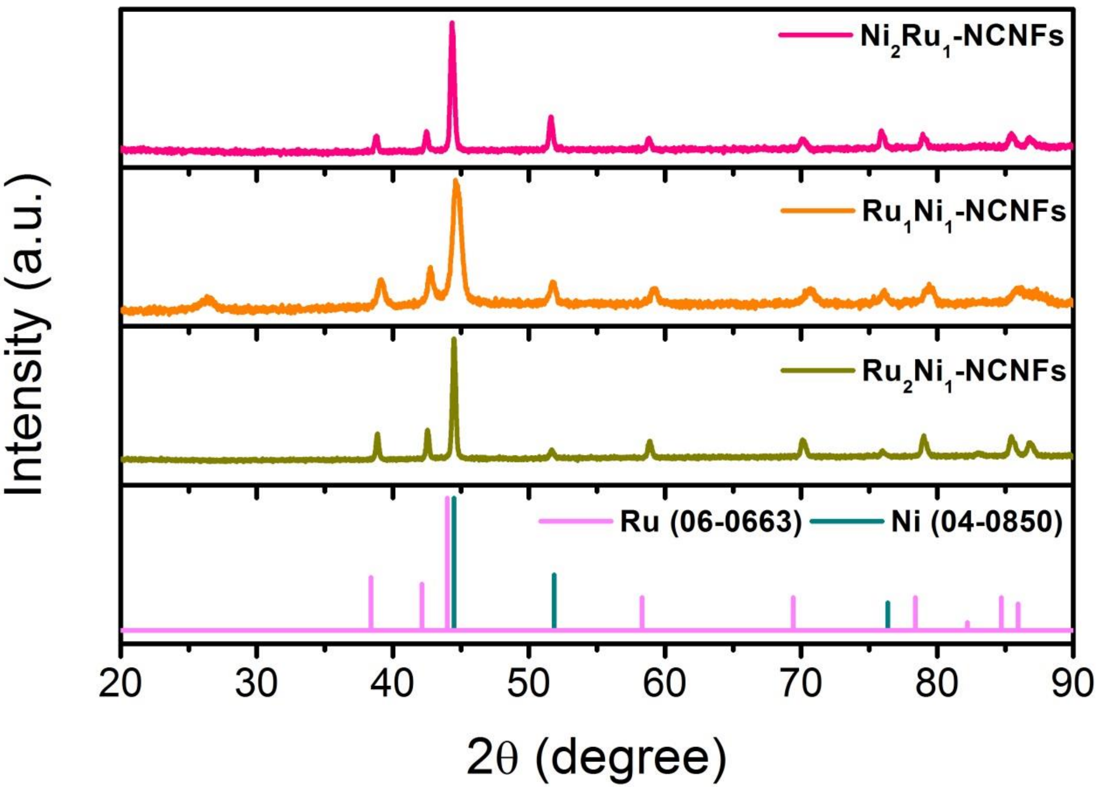

Figure S6. XRD patterns of Ru_1Ni_1-NCNFs, Ru_2Ni_1-NCNFs and Ni_2Ru_1-NCNFs.

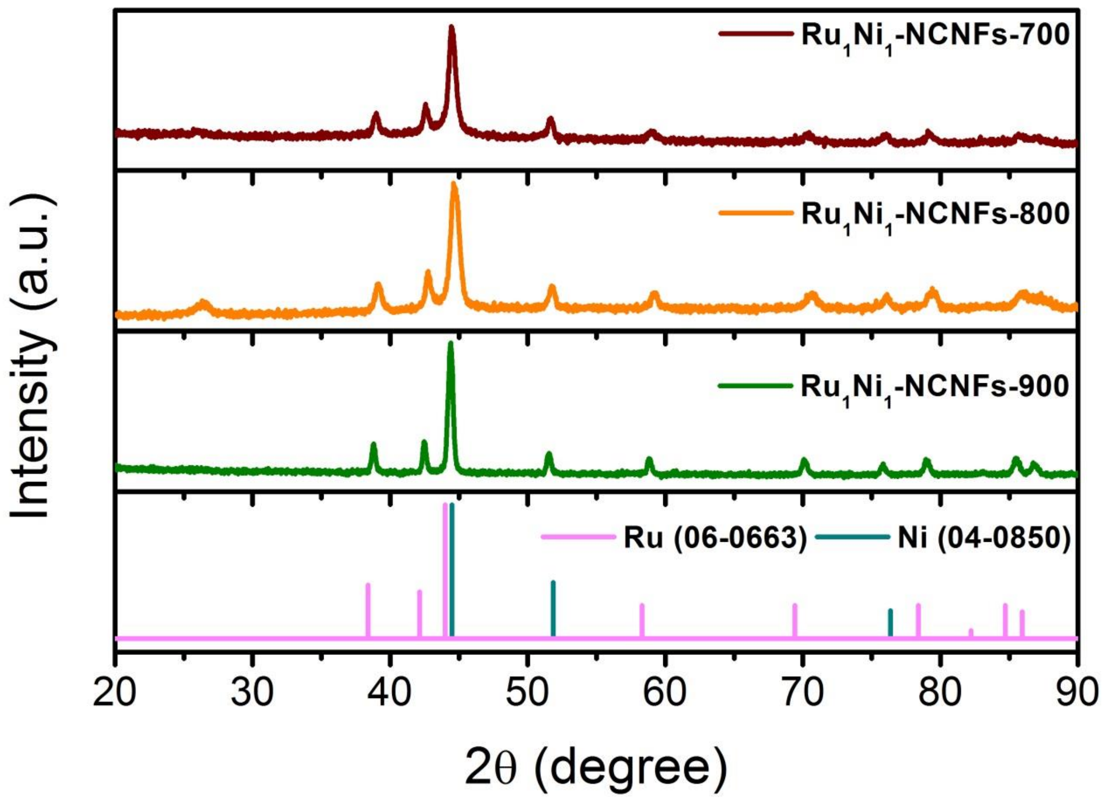

Figure S7. XRD patterns of Ru_1Ni_1-NCNFs-700, Ru_1Ni_1-NCNFs-800 and Ru_1Ni_1-NCNFs-900.

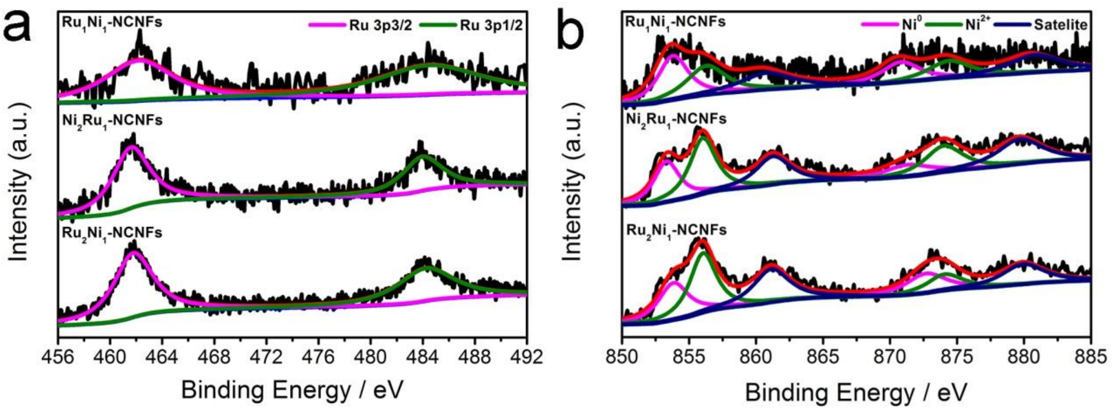

Figure S8. (a) Ru 3p XPS spectra of Ru_1Ni_1-NCNFs, Ni_2Ru_1-NCNFs and Ru_2Ni_1-NCNFs. (b) Ni 2p XPS spectra of Ru_1Ni_1-NCNFs, Ni_2Ru_1-NCNFs and Ru_2Ni_1-NCNFs.

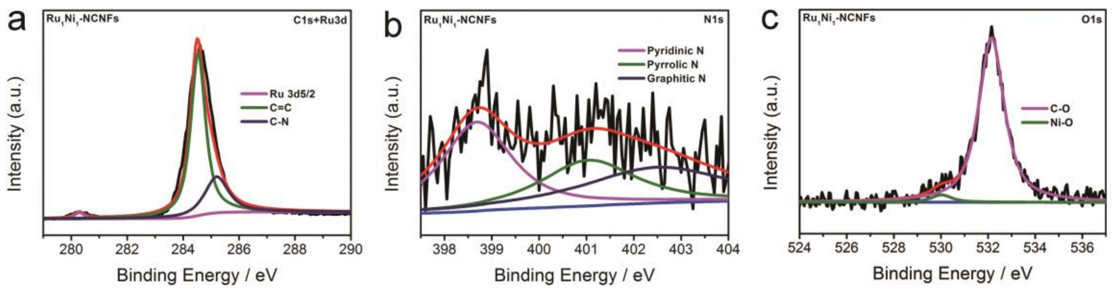

Figure S9. XPS spectra of the prepared Ru_1Ni_1-NCNFs: (a) C 1S, (b) N 1s and (c) O 1s.

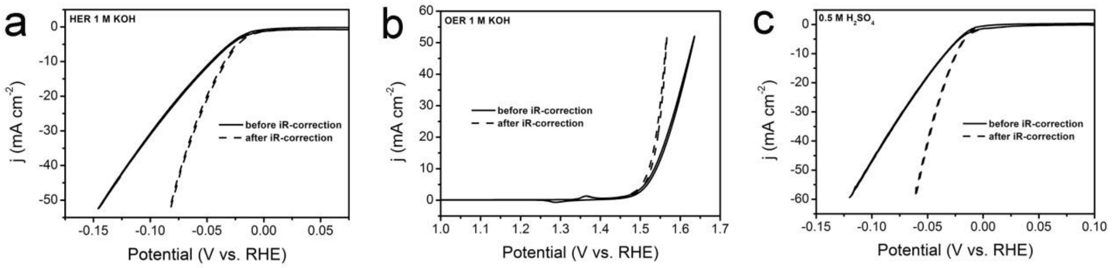

Figure S10. (a) HER CV curves of the Ru_1Ni_1-NCNFs without or with iR compensation in 1 M KOH at a scan rate of 1 mV s^{-1}. (b) OER CV curves of the Ru_1Ni_1-NCNFs without or with iR compensation in 1 M KOH at a scan rate of 1 mV s^{-1}. (c) HER CV curves of the Ru_1Ni_1-NCNFs without or with iR compensation in 0.5 M H_2SO_4 at a scan rate of 1 mV s^{-1}.

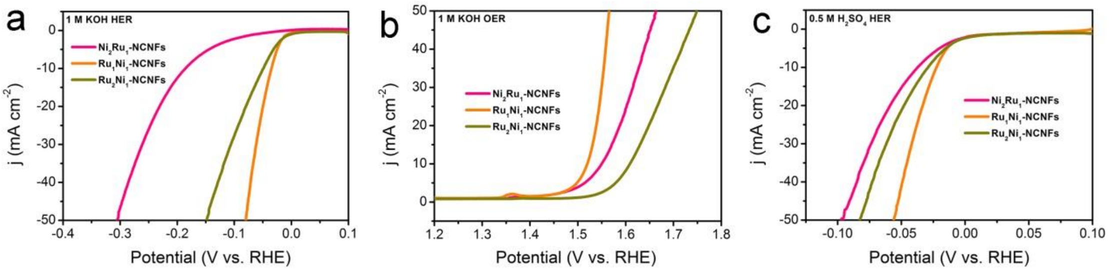

Figure S11. (a) The HER polarization curves of the as-prepared Ru_1Ni_1-NCNFs, Ru_2Ni_1-NCNFs and Ni_2Ru_1-NCNFs with iR compensation in 1 M KOH at a scan rate of 1 mV s^{-1}.(b) The OER polarization curves of the as-prepared Ru_1Ni_1-NCNFs, Ru_2Ni_1-NCNFs and Ni_2Ru_1-NCNFs with iR compensation in 1 M KOH at a scan rate of 1 mV s^{-1}. (C) The HER polarization curves of the as-prepared Ru_1Ni_1-NCNFs, Ru_2Ni_1-NCNFs and Ni_2Ru_1-NCNFs with iR compensation in 0.5 M H_2SO_4 at a scan rate of 1 mV s^{-1}.

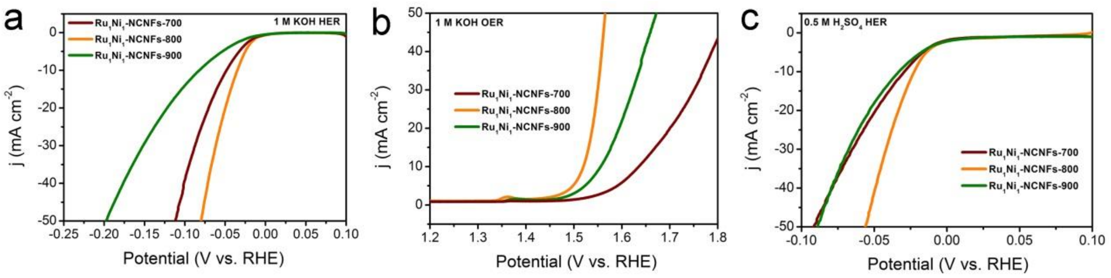

Figure S12. (a) The HER polarization curves of the as-prepared Ru_1Ni_1-NCNFs-700, Ru_1Ni_1-NCNFs-800and Ru_1Ni_1-NCNFs-900 with iR compensation in 1 M KOH at a scan rate of 1 mV s^{-1}.(b) The OER polarization curves of the as-prepared Ru_1Ni_1-NCNFs-700, Ru_1Ni_1-NCNFs-800 and Ru_1Ni_1-NCNFs-900 with iR compensation in 1 M KOH at a scan rate of 1 mV s^{-1}. (C) The HER polarization curves of the as-prepared Ru_1Ni_1-NCNFs-700, Ru_1Ni_1-NCNFs-800 and Ru_1Ni_1-NCNFs-900 with iR compensation in 0.5 M H_2SO_4 at a scan rate of 1 mV s^{-1}.

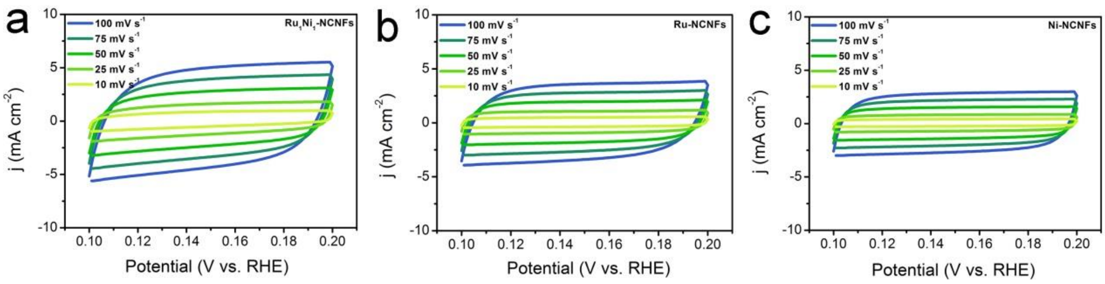

Figure S13. CV curves of (a) Ru_1Ni_1-NCNFs, (b) Ru-NCNFs and (c) Ni-NCNFs at different scan rates (10 mV s^{-1} -100 mV s^{-1}).

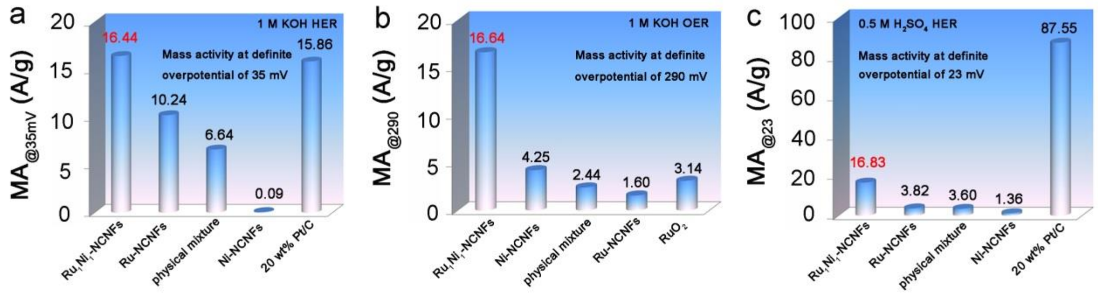

Figure S14. Mass activity at fixed overpotential of (a) 35 mV (1 M KOH HER), (b) 290 mV (1 M KOH OER) and (c) 23 mV (0.5 M H_2SO_4).

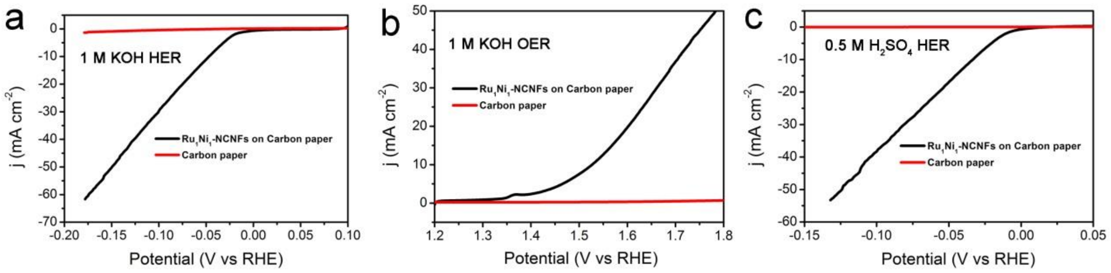

Figure S15. (a) HER and (b) OER polarization curves of Ru_1Ni_1-NCNFs catalyst without iR compensation with a loading of 2.5 mg cm^{-2} loaded on carbon paper electrode in 1 M KOH at a scan rate of 1 mV s^{-1}. (c) HER polarization curves of Ru_1Ni_1-NCNFs catalyst without iR compensation with a loading of 2.5 mg cm^{-2} loaded on carbon paper electrode in 0.5 M H_2SO_4 at a scan rate of 1 mV s^{-1}.

Note: Ru_1Ni_1-NCNFs catalyst supported on carbon paper requires η of 47 mV for HER and 298 mV for OER to achieve a current density of 10 mA cm^{-2} in 1 M KOH and needs 34 mV for HER to drive a current density of 10 mA cm^{-2} in 0.5 M H_2SO_4, while the bare carbon paper has negligible electrocatalytic activity.

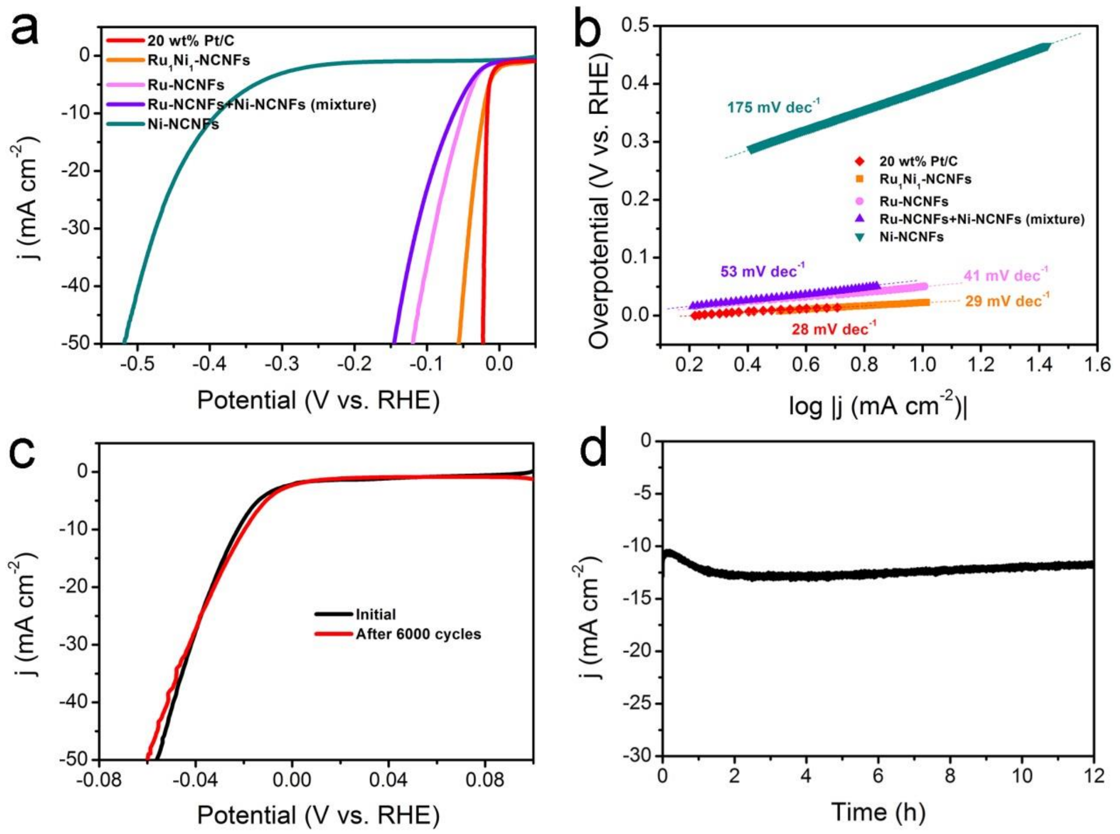

Figure S16. HER tests in 0.5 M H_2SO_4. (a) The HER polarization curves for as-prepared catalysts at a scan rate of 1 mV s^{-1}. (b) Tafel plots and exchange currents for different catalysts. (c) Polarization curves before and after 6000 CV cycles. The mass loading is 0.612 mg cm^{-2} loaded on RDE for the measurements in (a-e). (d) The chronoamperometric test (without iR-drop compensation) was conducted under a constant overpotential of 40 mV for Ru_1Ni_1-NCNFs on carbon paper with a loading of 2.5 mg cm^{-2}.

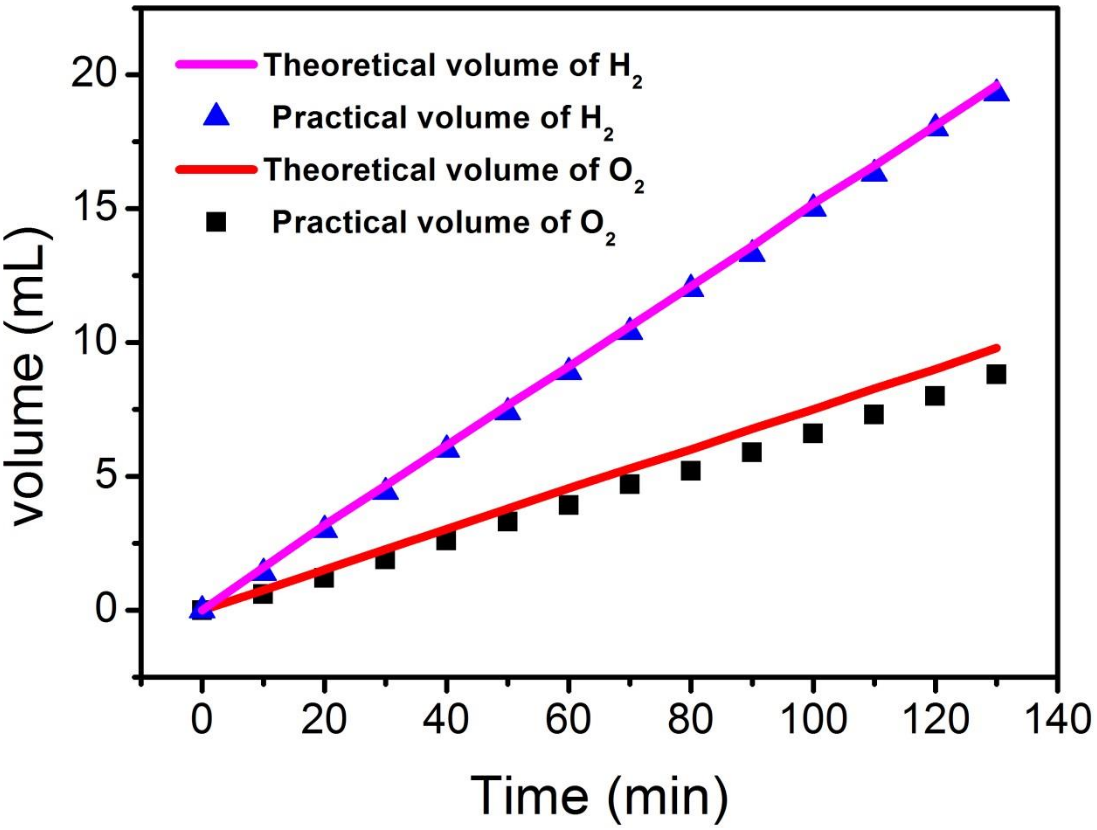

Figure S17. Related Faraday efficiency of Ru_1Ni_1-NCNFs for overall water splitting.

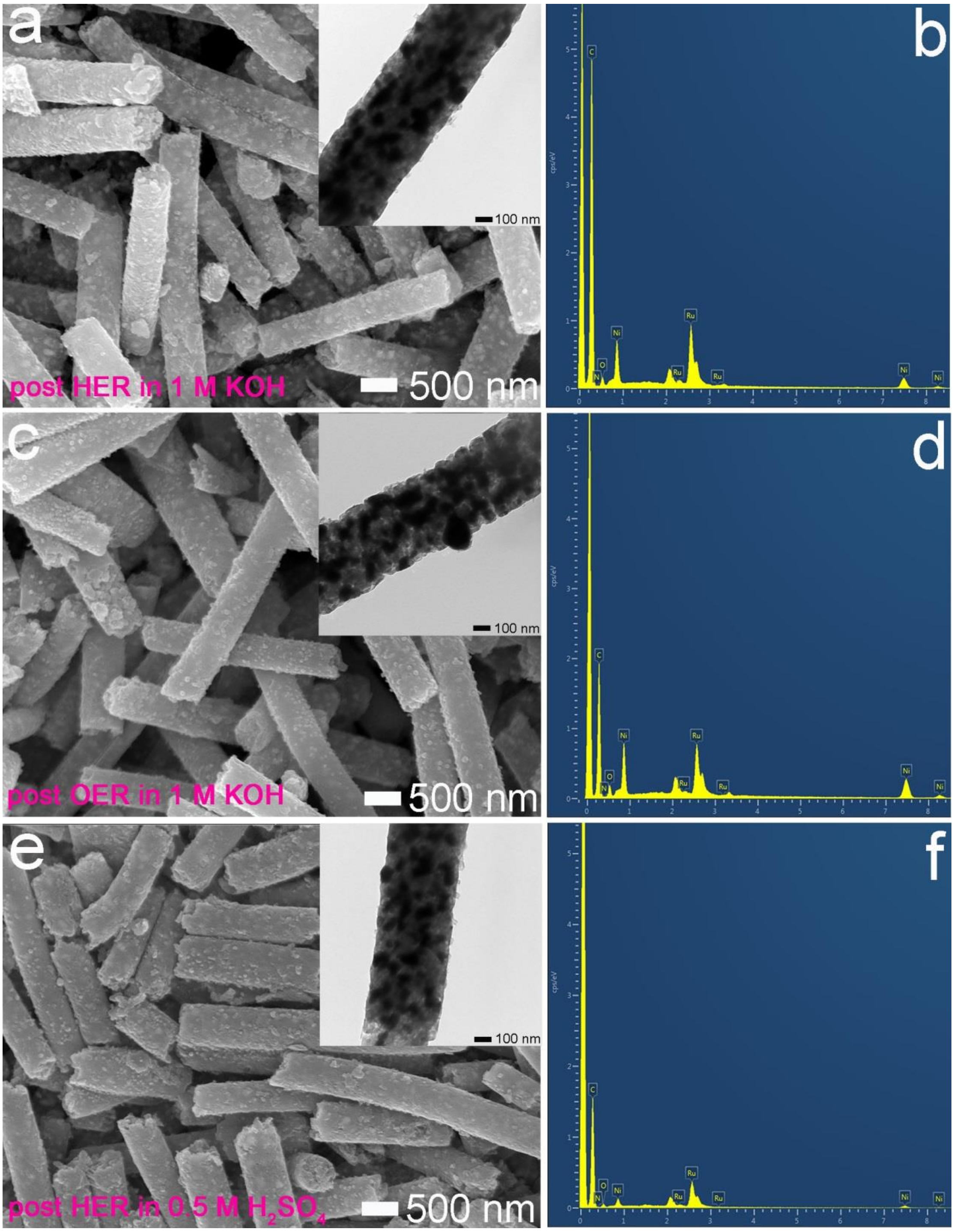

Figure S18. SEM and corresponding inset TEM images of Ru_1Ni_1-NCNFs (a) after HER stability test in 1 M KOH, (c) after OER stability test in 1 M KOH and (e) after HER stability test in 0.5 M H_2SO_4. (b, d and f) Corresponding EDX spectra of Ru_1Ni_1-NCNFs after stability tests.

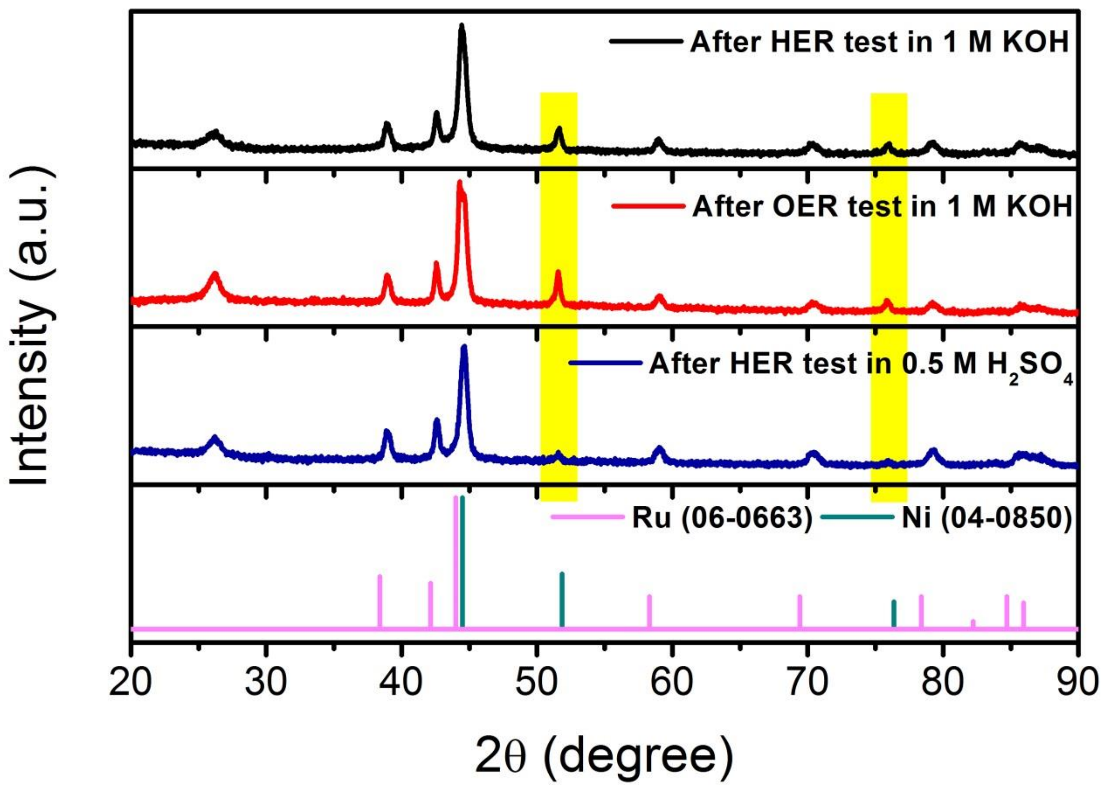

Figure S19. XRD characterization of Ru_1Ni_1-NCNFs catalyst after long-term stability tests.

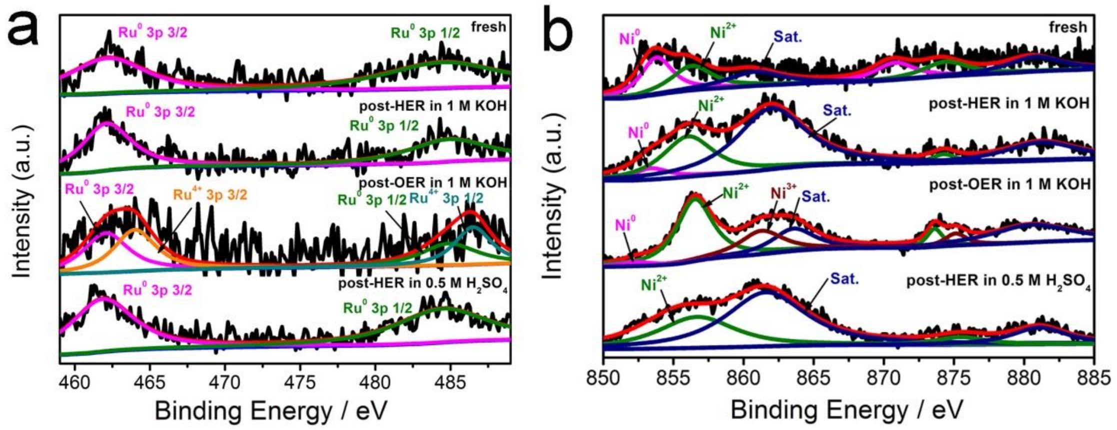

Figure S20. XPS characterization of Ru_1Ni_1-NCNFs catalyst after stability tests. (a) High-resolution Ru 3p XPS spectra. (b) High-resolution Ni 2p XPS spectra.

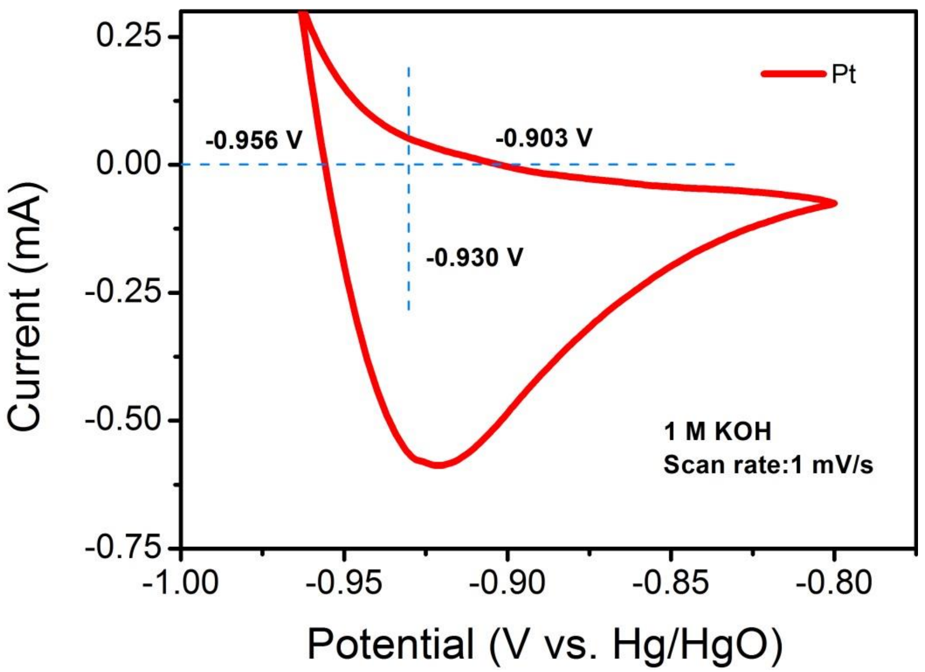

Figure S21. RHE voltage calibration.

Table S1. Comparison of the TOF values for HER in alkaline electrolyte of our catalysts with recently reported ones.

Electrocatalyst	TOF (s^{-1}) for HER	Reference
Ru_1Ni_1-NCNFs	0.076 at 80 mV	This work
δ-WN/$Co_{2.45}$	0.0193 at 200 mV	J. Mater. Chem. A 2018, 6, 10967.
$NiCo_2P_x$	0.056 at 100 mV	Adv. Mater. 2017, 29, 1605502.
P-doped Mo_2C@C nanospheres	0.00371 at 10 mV	ACS Nano 2016, 10, 8851.
γ-Mo_2N	0.07 at 250 mV	J. Mater. Chem. A 2015, 3, 8361.
Ni_2P (pellet)	0.014 at 200 mV	Energy Environ. Sci. 2015, 8, 1027.
Ni-Mo	0.05 at 100 mV	ACS Catal. 2013, 3, 166.

Table S2. Comparison of the HER activity for Ru_1Ni_1-NCNFs electrocatalyst with other reported electrocatalysts in alkaline solution.

Catalyst	Electrolyte	Overpotential at 10 mA cm^{-2} (mV)	Tafel slope (mV $decade^{-1}$)	Reference
Ru_1Ni_1-NCNFs	1 M KOH	35	30	This work
$Pd_{50}Ru_{50}$/CNs	0.1 M KOH	37.3	67.9	Catalysts 2018, 8, 329
Cu_{2-x}@RuNPS	1 M KOH	82	48	Small 2017, 13, 1700052
Ru/CP	1 M KOH	35	50	Nanoscale 2017, 9, 16616.
Ru/MoS_2/CP	1 M KOH	13	60	Nanoscale 2017, 9, 16616.
Ru@C_2N	1 M KOH	17	38	Nat. Nanotechnol. 2017, 12, 441.
Ru/C_3N_4/C	1 M KOH	79	-	J. Am. Chem. Soc. 2016, 138, 16174.
RuP_2@NPC	1 M KOH	52	69	Angew. Chem. Int. Ed. 2017, 56, 11559.
1D-RuO_2-CN_x	1 M KOH	63	-	ACS Appl. Mater. Interfaces 2016, 8, 28678.
NiO/Ru@Ni	1 M KOH	39	75	J. Mater. Chem. A 2019, 7, 2344.
Ru_3Ni_3 NAs	1 M KOH	39	26.9	iScience 2019, 11, 492.
β-$Ni(OH)_2$/Pt	1 M KOH	115	42	ACS Energy Lett. 2018, 3, 237.
NiO/Ni-CNT	1 M KOH	85	82	Nat. Commun. 2014, 5, 4695.
NiSe/Ni foam	1 M KOH	96	120	Angew. Chem. Int. Ed. 2015, 54, 9351.
NiP_2 NS/CC	1 M KOH	102	65	Nanoscale 2014, 6, 13440.
MoS_2/Ni_3S_2	1 M KOH	110	83	Angew. Chem. Int. Ed. 2016, 55, 6702.
NiCu@C	1 M KOH	74	94	Adv. Energy Mater. 2017, 8, 1701759.
$Ni(OH)_2$ nanosheets	1 M KOH	80	70	Chem 2017, 3, 122.
$Ni(OH)_2$@CuS	1 M KOH	95	104	Nano Energy 2018, 44, 7.
V-doped NiS_2 nanosheets	1 M KOH	110	90	ACS Nano 2017, 11, 11574.
MoS_2-Ni_3S_2 heterostructures	1 M KOH	110	83	Angew. Chem. Int. Ed. 2016, 55, 6701.

Table S3. Comparison of the TOF values for HER in acidic electrolyte of our catalysts with recently reported ones.

Electrocatalyst	TOF (s^{-1}) for HER	Reference
Ru_1Ni_1-NCNFs	0.074 at 55 mV	This work
$Ni_{0.33}Co_{0.67}Se_2$	0.058 at 100 mV	Adv. Energy Mater. 2017, 7, 1602089.
Au NPs (10 nm)/C	0.000443 at 350 mV	Electrochimica Acta 2016, 209, 440.
Co-NG	0.022 at 50 mV	Nat. Commun. 2015, 6, 8668.
γ-Mo_2N	0.03 at 250 mV	J. Mater. Chem. A 2015, 3, 8361.
CoP/Ti	0.046 at 100 mV	Angew. Chem. Int. Ed. 2014, 19, 5427.
Ni_2P	0.015 at 100 mV	J. Am. Chem. Soc. 2013, 135, 9267.

Table S4. Comparison of the HER activity for Ru_1Ni_1-NCNFs electrocatalyst with other reported electrocatalysts in acidic solution.

Catalyst	Electrolyte	Overpotential at 10 mA cm^{-2} (mV)	Tafel slope (mV $decade^{-1}$)	Reference
Ru_1Ni_1-NCNFs	0.5 M H_2SO_4	23	29	This work
$Pd_{50}Ru_{50}$/CNs	0.5 M H_2SO_4	45.1	67.6	Catalysts 2018, 8, 329
Cu_{2-x}@RuNPS	0.5 M H_2SO_4	129	51	Small 2017, 13, 1700052
Ru@C_2N	0.5 M H_2SO_4	135	30	Nat. Nanotechnol. 2017, 12, 441.
Ru/CP	0.5 M H_2SO_4	78	-	Nanoscale 2017, 9, 16616.
Ru/MoS_2/CP	0.5 M H_2SO_4	96	-	Nanoscale 2017, 9, 16616.
Ru/GLC	0.5 M H_2SO_4	35	46	ACS Appl. Mater. Interfaces 2016, 8, 35132.
Ru/SiNW-42.9	0.5 M H_2SO_4	200	81	Electrochem. Commun. 2015, 52, 29.
GCE-S-GNs-1000-CB-Ru	0.5 M H_2SO_4	75	61	Carbon 2015, 93, 762.
Co-Ru-Pt	0.5 M H_2SO_4	73.1@4 mA cm^{-2}	30.4	Int. J. Hydrogen Energy 2017, 42, 38.
RuP_2@NPC	0.5 M H_2SO_4	38	38	Angew. Chem. Int. Ed. 2017, 56, 11559.
1D-RuO_2-CN_x	0.5 M H_2SO_4	93	40	ACS Appl. Mater. Interfaces 2016, 8, 28678.
Ru_3Ni_3 NAs	0.5 M H_2SO_4	39	53.9	iScience 2019, 11, 492.
Ru/C_3N_4/C	0.5 M H_2SO_4	~75	-	J. Am. Chem. Soc., 2016, 138, 16174.
Ru-MoO_2	0.5 M H_2SO_4	55	44	J. Mater. Chem. A 2017, 5, 5475.
RuO_2 Nanorods	0.5 M H_2SO_4	130	61.26	Colloids Surf., A 2019, 560, 141.
NiP_2 NS/CC	0.5 M H_2SO_4	75	51	Nanoscale 2014, 6, 13440.
$NiCo_2P_x$/CF	0.5 M H_2SO_4	104	59.6	Adv. Mater. 2017, 1605502.

Table S5. Comparison of the TOF values for OER in alkaline electrolyte of our catalysts with recently reported ones.

Electrocatalyst	TOF (s^{-1}) for OER	Reference
Ru_1Ni_1-NCNFs	0.038 at 335 mV	This work
Co-Ni_3N	0.0134 at 350 mV	Adv. Mater. 2018, 30, 1705516.
$Cu_3Mo_2O_9$/NF	0.02 at 300 mV	Inorganic chemistry 2018, 57, 1220.
Co4Ni1P NTs	0.032 at 300 mV	Adv. Funct. Mater. 2017, 27, 1703455.
NiCo@$NiCoO_2$/C PMRAs	0.022 at 450 mV	Adv. Mater. 2018, 30, 1705442.
Ni-MnO_2	0.0356 at 450 mV	ChemCatChem 2019, 11, 1.
Co_9S_8@NOSC-900	0.0129 at 340 mV	Adv. Funct. Mater. 2017, 27, 1606585

Table S6. Comparison of the OER activity for Ru_1Ni_1-NCNFs electrocatalyst with other reported electrocatalysts in alkaline solution.

Catalyst	Electrolyte	Overpotential at 10 mA cm^{-2} (mV)	Reference
Ru_1Ni_1-NCNFs	1 M KOH	290	This work
Ru_3Ni_3 NAs	1 M KOH	304	iScience 2019, 11, 492.
Ru	1 M KOH	320	J. Am. Chem. Soc. 2013, 135, 16977.
Ir	1 M KOH	430	J. Am. Chem. Soc. 2015, 137, 4347.
Ru-RuP_x-Co_xP	0.1 M KOH	291	Nano Energy 2018, 53, 270.
Ag@Co_xP	1 M KOH	310	ACS Catal. 2017, 7, 7038.
Exfoliated NiFe LDH	1 M KOH	302	Nat. Commun. 2014, 5, 4477.
Co_3O_4/$NiCo_2O_4$	1 M KOH	340	J. Am. Chem. Soc. 2015, 137, 5590.
NiMo-PVP/NiFe-PVP	1 M KOH	297	Adv. Energy Mater. 2017, 1700220.
Ni-Mo nitride	1 M KOH	295	J. Mater. Chem. A 2017, 13648.
PO-Ni/Ni-N-CNFs	1 M KOH	420	Nano Energy 2018, 51, 286.
$Ni_{1-y-z}Fe_yCr_zO_x$	1 M KOH	320	Sci. Rep. 2017, 7, 44192.
$Ni1Fe_2$-250	1 M KOH	310	Chemcatchem 2016, 8, 992.
$NiCo_2O_4$	1 M KOH	290	Angew. Chem. Int. Ed. 2016, 55, 6290.
NiFe-LDH/$NiCo_2O_4$/NF	1 M KOH	290	ACS Appl. Mater. Interfaces 2017, 9, 1488.
NiCo/$NiCoO_X$	1 M KOH	361	ACS Appl. Mater. Interfaces 2016, 8, 3208.
3D Ni-NG	1 M KOH	320	Energy Environ. Sci. 2013, 6, 3693.
$NiCo_2O_4$ Nanowire film	1 M KOH	320	Nano Energy 2015, 11, 333.
$Ni(OH)_2$/NF	1 M KOH	350	Chem. Phys. Lett. 2016, 644, 51.
NiO@Ni@Carbon fiber	1 M KOH	300	ACS Sustain. Chem. Eng. 2017, 5, 529.
Ni_5P_4/Ni foil	1 M KOH	290	Angew. Chem. Int. Ed. 2015, 54, 12361.
NiCo LDH	1 M KOH	367	Nano Lett. 2015, 15, 1421.
$NiCo_{2.7}(OH)_x$	1 M KOH	350	Adv. Energy Mater. 2015, 5, 1402031.
Co-Ni_3N	1 M KOH	307	Adv. Mater. 2018, 30, 1705516

Table S7. Comparison of the overall water splitting activity of the Ru_1Ni_1-NCNFs electrocatalyst with other reported electrolyzers in alkaline solution.

Catalyst	Electrolyte	Voltage at 10 mA cm^{-2} (V)	Reference
Ru_1Ni_1-NCNFs	1 M KOH	1.564	This work
Ni-Mo nitride	1 M KOH	1.596	J. Mater. Chem. A 2017, 13648.
NiSe	1 M KOH	1.63	Angew Chem Int Ed. 2015, 54, 9351.
Ni_2P	1 M KOH	1.63	Energy Environ Sci. 2015, 8, 2347.
PO-Ni/Ni-N-CNFs	1 M KOH	1.69	Nano Energy 2018, 51, 286.
NiO_{OER} // Ni_2P_{HER}	1 M KOH	1.65	Nanoscale 2017, 9, 4409.
$Ni_{0.9}Fe_{0.1}$/NC-NF	1 M KOH	1.58	ACS Catal. 2015, 6, 580.
NiFeLDH@NiCoP/NF	1 M KOH	1.57	Adv. Funct. Mater. 2018, 28, 1706847.
Ni_8P_3/Ni	1 M KOH	1.61	Adv. Funct. Mater. 2016, 26, 3314.
Ni-Fe-P-350	1 M KOH	1.68	ACS Appl. Mater. Interfaces 2017, 9, 26134.
TiN@Ni_3N	1 M KOH	1.64	J Mater Chem A 2016, 4, 5713.
NiMo-PVP‖NiFe-PVP	1 M KOH	1.66	Adv. Energy Mater. 2017, 7, 1700220.
NiMoN‖CoN	1 M KOH	1.63	Angew Chem Int Ed. 2016, 55, 8670.
$CoNi(OH)_x$‖NiN_x	1 M KOH	1.65	Adv Energy Mater 2016, 6, 1501661.
EG/$Co_{0.85}$Se/NiFe-LDH	1 M KOH	1.67	Energy Environ. Sci. 2016, 9, 478.
$NiCo_2S_4$/carbon cloth	1 M KOH	1.68	Nanoscale 2018, 7, 15122.
NiMo HNRs/Ti mesh	1 M KOH	1.64	J. Mater. Chem. A 2015, 3, 20056.
$Ni_3ZnC_{0.7}$-550/NF	1 M KOH	1.65	J. Am. Chem. Soc. 2014, 136, 7587.
NiCoP/Ti	1 M KOH	1.64	J. Mater. Chem. A 2017, 5, 10561.
$NiCo_2P_x$/CNTs	1 M KOH	1.61	J. Mater. Chem. A 2018, 6, 7420.
NiFe/$NiCo_2O_4$/Ni Foam	1 M KOH	1.67	Adv. Funct. Mater. 2016, 26, 3515.
Ni@NC-800/NF	1 M KOH	1.60	J. Mater. Chem. A 2016, 4, 5639.
